# Complex Interplay Between MAZR and Runx3 Regulates the Generation of Cytotoxic T Lymphocyte and Memory T Cells

**DOI:** 10.3389/fimmu.2021.535039

**Published:** 2021-03-17

**Authors:** Alexandra Franziska Gülich, Ramona Rica, Caroline Tizian, Csilla Viczenczova, Kseniya Khamina, Thomas Faux, Daniela Hainberger, Thomas Penz, Remy Bosselut, Christoph Bock, Asta Laiho, Laura L. Elo, Andreas Bergthaler, Wilfried Ellmeier, Shinya Sakaguchi

**Affiliations:** ^1^ Division of Immunobiology, Institute of Immunology, Center for Pathophysiology, Infectiology and Immunology, Medical University of Vienna, Vienna, Austria; ^2^ CeMM Research Center for Molecular Medicine of the Austrian Academy of Sciences, Vienna, Austria; ^3^ Turku Bioscience Centre, University of Turku and Åbo Akademi University, Turku, Finland; ^4^ Institute of Artificial Intelligence and Decision Support, Center for Medical Statistics, Informatics, and Intelligent Systems, Medical University of Vienna, Vienna, Austria; ^5^ Laboratory of Immune Cell Biology, Center for Cancer Research, National Cancer Institute, National Institutes of Health, Bethesda, MD, United States

**Keywords:** MAZR/PATZ1, Runx3, cytotoxic T lymphocyte, transcriptional control, LCMV, memory T cell

## Abstract

The BTB zinc finger transcription factor MAZR (also known as PATZ1) controls, partially in synergy with the transcription factor Runx3, the development of CD8 lineage T cells. Here we explored the role of MAZR as well as combined activities of MAZR/Runx3 during cytotoxic T lymphocyte (CTL) and memory CD8^+^ T cell differentiation. In contrast to the essential role of Runx3 for CTL effector function, the deletion of MAZR had a mild effect on the generation of CTLs *in vitro*. However, a transcriptome analysis demonstrated that the combined deletion of MAZR and Runx3 resulted in much more widespread downregulation of CTL signature genes compared to single Runx3 deletion, indicating that MAZR partially compensates for loss of Runx3 in CTLs. Moreover, in line with the findings made *in vitro*, the analysis of CTL responses to LCMV infection revealed that MAZR and Runx3 cooperatively regulate the expression of CD8α, Granzyme B and perforin *in vivo*. Interestingly, while memory T cell differentiation is severely impaired in Runx3-deficient mice, the deletion of MAZR leads to an enlargement of the long-lived memory subset and also partially restored the differentiation defect caused by loss of Runx3. This indicates distinct functions of MAZR and Runx3 in the generation of memory T cell subsets, which is in contrast to their cooperative roles in CTLs. Together, our study demonstrates complex interplay between MAZR and Runx3 during CTL and memory T cell differentiation, and provides further insight into the molecular mechanisms underlying the establishment of CTL and memory T cell pools.

## Introduction

CD8^+^ T cells play a central role during immune responses against viruses, intracellular bacteria and protozoan parasites and are also key regulators of anti-tumor immunity (1–3). Upon activation, antigen-specific CD8^+^ T cells proliferate and differentiate into a heterogenous population of “armed” cytotoxic T lymphocytes (CTLs), which release a large amount of cytolytic proteins as well as various inflammatory cytokines (1–3). While the majority of CTLs undergo apoptosis after the clearance of pathogens or tumors, a fraction of the cells is programmed for long-term survival and becomes memory T cells, which consist of various subsets distinguished by phenotypes, functions and locations (4–7). During the last two decades, key transcriptional regulators controlling CTL and memory T cell differentiation have been identified, leading to an understanding of the basic transcriptional networks of the differentiation processes (8–10). However, the gene regulatory networks connecting cooperative as well as antagonistic interplay between these transcription factors are still not fully understood.

Myc-associated zinc finger-related factor (MAZR; also known as PATZ1 and encoded by the *Patz1* gene) has been identified as an important transcriptional regulator controlling the development of CD4^+^ regulatory and CD8^+^ T cells (11–14). MAZR belongs to the broad-complex, tramtrack and bric-à-brac (BTB) and zinc finger (ZF) containing transcription factor family, a family known to function both as transcriptional repressors and activators in a context-dependent manner (15, 16). In double-negative (DN) thymocytes MAZR binds to multiple *Cd8* enhancer regions and thereby negatively regulates CD8 expression (11). In addition, MAZR promotes cytotoxic lineage differentiation of MHC class I-selected double-positive (DP) thymocytes *via* repressing the expression of T-helper-inducing POZ/Krueppel-like factor (ThPOK, encoded by the *Zbtb7b* gene) (12). Further, MAZR is required to maintain ThPOK repression also in CD8^+^ T cells (13). MAZR interacts with Runt-related transcription factor (Runx) proteins (i.e. Runx1 and Runx3) and MAZR/Runx1 and MAZR/Runx3 complexes synergistically repress ThPOK expression in pre-selected DP thymocytes and CD8^+^ T cells, respectively (13). Thus, these studies identified MAZR as an important regulator of CD8 lineage differentiation, which acts in synergy with Runx proteins. However, the role of MAZR during CTL and memory T cell differentiation remains unexplored. Since recent studies have demonstrated an essential role of Runx3 for CTL function and the generation of memory T cell precursors during LCMV infection (17–19), we hypothesized that an interplay between MAZR and Runx3 controls these processes. In the present study we examined the impact of loss of MAZR and/or Runx3 on CTL and memory T cell differentiation by generating and analyzing T cell lineage- as well as cytotoxic lineage-specific MAZR-, Runx3- and MAZR/Runx3 double-deficient mice. Our study demonstrates that, while MAZR plays a compensatory role for Runx3-mediated transcriptional programs in CTLs, the two molecules exhibit distinct functions in the generation of memory T cell subsets. This suggests the differentiation stage-specific interplay between MAZR and Runx3, and thereby provides further insight into the transcriptional mechanisms governing the differentiation process of naïve CD8^+^ T cells into CTLs and memory T cells.

## Methods

### Mice

*Mazr* ^f/f^ (20), *Runx3* ^f/f^ (21), *Thpok* ^f/f^ (22), *Thpok*-GFP knock-in (23), *Rosa26*-YFP reporter (24), *Cd4*-Cre (25) and *E8I*-Cre (26) mice have been described previously. Mice used for experiments were 8-12 weeks old, and were maintained in the preclinical research facility of the Medical University of Vienna. All animal experiments were evaluated by the ethics committee of the Medical University of Vienna and approved by the Federal Ministry for Science, Research and Economy, Vienna, Austria. Animal husbandry and experimentation was performed under the national laws (Federal Ministry for Science, Research and Economy, Vienna, Austria) and according to the guidelines of the Federation of European Laboratory Animal Science Associations (FELASA), which match that of Animal Research: Reporting of *In Vivo* Experiments (ARRIVE) from the National Centre for the Replacement, Refinement and Reduction of Animals in Research (London, UK).

### LCMV Infection

Mice were infected with 2 x 10^5^ PFU of the LCMV Armstrong strain intraperitoneally (i.p.) (27). Eight days after infection mice were euthanized, and the single cell suspensions of splenocytes were prepared as previously described (11). Virus-specific CD8^+^ T cells were identified with R-phycoerythrin (PE) or allophycocyanin (APC)-labeled H2-D^b^/GP33-41 and H2-D^b^/NP396-404 dextramers (Immudex) and brilliant violet (BV) 421-labled H2-D^b^/GP33-41 tetramers (kindly provided by the NIH Tetramer Core Facility). For the detection of cytokine production, splenocytes were incubated with 1 μM GP33 (KAVYNFATC) peptides (Anaspec) in the presence of GolgiStop (BD Biosciences) and GolgiPlug (BD Biosciences) for 4.5 hours at 37°C. In order to detect CD107a and CD107b surface externalization, anti-CD107a (1:1000; Biolegend) and anti-CD107b (1:1000; Biolegend) antibodies were added during peptide stimulation.

### Isolation and Activation of CD8^+^ T Cells

Splenic T cells were enriched by negative depletion before cell sorting. In brief, after red blood cell lysis, splenocytes (5-10 x 10^7^ cells) were incubated with biotinylated (bio)-anti-Gr1 (RB6-8C5, final concentration 4 µg/ml), bio-anti-CD45R (RA3-6B2, 4 µg/ml), bio-anti-Ter119 (Ter119, 1 µg/ml), bio-anti-NK1.1 (PK136, 1 µg/ml), bio-anti-CD11b (M1/70, 1 µg/ml), bio-anti-CD11c (HL3, 1 µg/ml) in 0.5 ml PBS/2% FCS for 30 min at ice. The biotinylated antibodies were purchased from Biolegend and BD Biosciences. Subsequently, cells were washed and purified by negative depletion using MagniSort Streptavidin Negative Selection Beads (Thermo Fisher) according to the manufacturer´s protocol. Enriched T cells were sorted with a FACS Aria I cell sorter (BD Biosciences) or a SH800S Cell Sorter (Sony Biotechnology) for CD4^–^CD8α^+^CD62L^+^CD44^–^ population (For Runx3- and MAZR/Runx3 double-deficient mice, CD4^mid-high^CD8α^+^CD62L^+^CD44^–^ population was included for the sorting). Sorted naïve CD8^+^ T cells were stimulated with plate-bound anti-CD3 (145-2C11, 2 µg/ml; BD Biosciences) and anti-CD28 (2 μg/ml, BD Biosciences) on 48-well plates (0.3 – 0.5 x 10^6^ cells/well) in 1 ml of complete RPMI1640 medium (Sigma, supplemented with 10% FCS (Sigma), 100 U/ml penicillin-streptomycin (GE Healthcare), 2 mM L-glutamin (Sigma), 0.1 mM non-essential amino acid (Lonza), 1 mM sodium pyruvate (GE Healthcare), 55 μM of β-mercaptoethanol (Sigma) in the presence of recombinant human interleukin 2 (rhIL-2, 20 U/ml; Peprotech). Two days after stimulation cells were split 1:2 to 3, placed in fresh T cell medium containing 100 U/ml rhIL-2 and thereafter split 1:2 to 3 every 2 days. For the generation of IL-12-treated CTLs, naïve CD8^+^ T cells were activated for two days as described above, and subsequently cultured in the presence of 100 U/ml rhIL-2 and 2 ng/ml IL-12 for three additional days (28). Cell proliferation was measured by labeling naïve T cells with 1 mM carboxyfluorescein diacetate succinimidyl ester (CFSE) (Thermo Fischer). Apoptotic cells were detected by Annexin V staining kit according to the manufacturer’s instruction (Thermo Fisher). In order to detect cytokine production, T cells were reactivated with ionomycin (500 ng/ml, Sigma) and phorbol 12-myristate 13-acetate (PMA, 50 ng/ml, Sigma) in the presence of GolgiStop (BD Biosciences) for 4.5 hours at 37°C.

### Retroviral Vector-Mediated Gene Transduction

Murine stem cell virus (MSCV)-based retroviral vectors containing the internal ribosome entry site (IRES)-GFP cassette were used for the overexpression of MAZR and distal promoter–derived Runx3 proteins (11, 29). Retroviral vectors (20 μg) were transiently transfected into Phoenix-E packaging cells plated in 10-cm culture dish by using standard calcium phosphate precipitation. One day after transfection, the medium was changed into complete RPMI 1640 medium. Viral supernatants were collected on the following day, filtrated through a 0.45-μm filter, and used for the infection of CD8^+^ T cells. Sorted naïve CD8^+^ T cells were stimulated with plate-bound anti-CD3 and anti-CD28 as described above. On the next day CD8^+^ T cells were suspended in 1 ml viral supernatants containing 10 μg polybrene (Sigma) and centrifuged at 600 g for 2 hours at 32 °C. After spin infection, cells were placed into 1 ml of fresh complete RPMI medium containing 20 U/ml rhIL-2. Cells were split 1:2 on the next day, cultured as described above and analyzed by a flow cytometer 6 days after activation.

### Antibodies and Flow Cytometry

Antibodies used in this study are listed in **Supplementary Table 1**. Foxp3/Transcription Factor Staining Buffer Set (Thermo Fischer) and BD Cytofix Fixation Buffer followed by BD Perm/Wash Buffer (BD Biosciences) were used for the intracellular staining of transcription factors and cytokines, respectively. Intracellular MAZR and Runx3 expression was detected by anti-MAZR/PATZ1 (D-5: Santa Cruz Biotechnology) and anti-Runx3 (R3-5G4: BD Biosciences) antibodies, followed by Alexa Fluor 647 anti-mouse IgG_1_ (RMG1-1: Biolegend) antibody. Flow cytometric data were collected with LSRII or Fortessa (BD), and analyzed with Flowjo software (BD Biosciences).

### *In Vitro* Cytotoxicity Assay

For the *in vitro* cytotoxicity assay, naïve CD8^+^ T cells were differentiated into CTLs as described above. Fc receptor–positive P815-GFP^+^ target cells (ATCC, TIB-64) were co-cultured with effector CD8^+^ T cells at 0.5:1, 1:1, 1:3 and 1:10 ratios in the presence of soluble anti-CD3 antibody at a total density of 2 x 10^5^ cells/200 μL/well in 96-well U-bottom shaped tissue culture plates. Cells were incubated for 4h at 37°C. After the incubation period, cells were harvested and cell suspension was stained for Annexin V and CD8α, and apoptosis of target cells (GFP^+^) was quantified by flow cytometry. As a negative control, effector and target cells were co-cultured without anti-CD3 antibody.

### RNA Isolation and Next Generation Sequencing

Three biological replicates from each genotype were prepared for RNA-sequencing. Total RNA of *in vitro* generated CTLs (6 days after activation) was isolated with RNeasy kit (Qiagen) combined with DNase I digestion in the extraction columns. RNA concentration was measured using Qubit 2.0 Fluorometric Quantitation (Life Technologies), and the RNA integrity number was determined using Experion Automated Electrophoresis System (Bio-Rad). RNA-seq libraries were prepared using a Sciclone NGS Workstation (PerkinElmer) and a Zepyhr NGS Workstation (PerkinElmer) with the TruSeq Stranded mRNA LT sample preparation kit (Illumina). Library amount and quality were determined using Qubit 2.0 Fluorometric Quantitation (Life Technologies) and Experion Automated Electrophoresis System (Bio-Rad). The libraries were sequenced by the Biomedical Sequencing Facility at CeMM using the Illumina HiSeq 3000/4000 platform and the 50-bp single-read configuration.

### Bioinformatic Analysis of RNA-Sequencing Data

The quality of the sequenced reads was checked using the FastQC tool (Babraham Bioinformatics, Babraham Institute, Cambridge, UK). STAR version 2.5.2b was used to align the reads to the mouse reference genome mm10, available at University of California, Santa Cruz Genome Bioinformatics Group (Illumina iGenomes website, San Diego, CA) (30). The number of uniquely mapped reads associated with each gene, according to RefSeq gene annotation, was counted using the Subread version 1.5.0 (31). The RNA-seq data are available from the Gene Expression Omnibus database (https://www.ncbi.nlm.nih.gov/geo; accession number GSE129772). The downstream analysis of the data was performed using R version 3.2.2 and its corresponding Bioconductor module 3.2. The count data were normalized using the trimmed mean of M-values method implemented in the edgeR R-package. The normalized data were further transformed using the voom approach in the limma R-package. R package limma was used for performing the statistical testing, and *p*-value < 0.05 and absolute fold change > 1.5 were required for detecting the differentially expressed (DE) genes between the sample groups. The *Gh* gene was removed from the final DE gene list as the inspection of the aligned reads revealed that the alignments for *Gh* had almost identical positions on a narrow region and contained many mismatches. The normalized expression values represented as Reads Per Kilobase per Million mapped reads (RPKM) were used as input for visualizations. Volcano plots and heat maps were generated with Prism 7 software (GraphPad). Venn diagrams were drawn with BioVenn web application (32). Enriched canonical pathways of the differentially expressed genes were predicted by using Ingenuity Pathway Analysis (Qiagen). To generate the list of the CTL signature genes, the gene expression pattern of vesicular stomatitis Indiana virus (VSV)-specific CTLs (8 days after infection) was compared with the one of naïve CD8^+^ T cells by utilizing the ImmGen database (33, 34), and top 200 upregulated genes and the additional *Cd8a* and *Cd8b1* genes were used to construct the list. Gene set enrichment analysis (GSEA) was performed with GSEA software from the Broad Institute (35).

### Quantitative Real-Time PCR

RNA isolation was performed using a RNeasy kit (Qiagen) according to the manufacturer’s instruction. To convert RNA samples into cDNA, samples were treated with SuperScript III reverse transcriptase and oligo(dT) primers according to the manufacturer’s protocol (Invitrogen). For quantitative real-time PCR SYBR Green (Bio-Rad) and the 7300 Real-Time PCR system was used. Primer sequences have been describer previously (29).

### Statistical Analysis

The statistical analyses were performed using Prism Software (GraphPad). For multiple comparisons in the majority of experiments, a one-way ANOVA analysis followed by Tukey’s multiple-comparison test was performed. The *p*-values were defined as following: *, *p* < 0.05; **, *p* < 0.01; ***, *p* < 0.001. For comparisons of mean fluorescence intensity (MFI) values *in vitro* experiments (where MFI levels of WT cells were set as 1), a one-sample *t*-test was conducted to compare MFI values between WT (i.e. 1) and each mutant group, whereas the values between three mutant groups were compared using a one-way ANOVA analysis as described above. The *p*-values based on a one-sample *t*-test (either *p* < 0.05, *p* < 0.01 or *p* < 0.001) were indicated above the respective diagrams. Finally, in the “overexpression” experiments (**Figure 2**) a paired two-tailed Student’s *t*-test was performed for comparisons of either MAZR- or Runx3-transduced mutant CTLs with control vector-transduced cells. The *p*-values were defined as described above. Differences that did not reach a statistically significant level (i.e. *p* ≥ 0.05) were not indicated.

## Results

### Combined Activities of MAZR and Runx3 Are Required for Appropriate CTL Differentiation *In Vitro*

**Figure 1:**
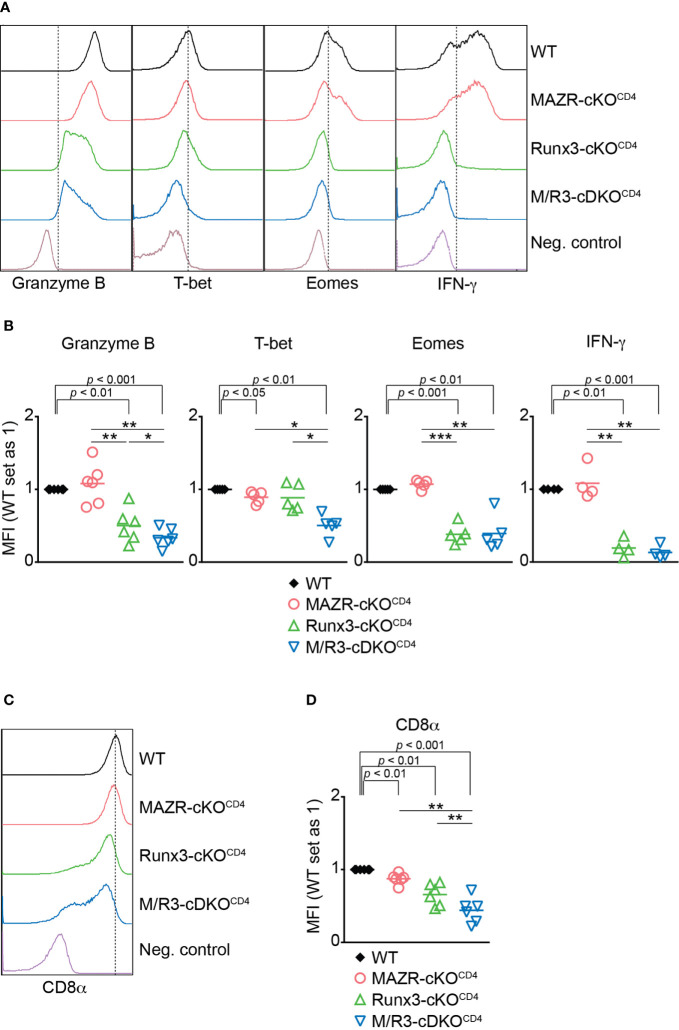
Combined activities of MAZR and Runx3 are required for appropriate CTL differentiation *in vitro*. **(A)** Histograms showing the expression of indicated proteins in the mutant CTLs (6 days after *in vitro* anti-CD3/28 stimulation). T helper 2 (Th2) cells as well as WT CTLs without restimulation were included as negative controls for the stainings of Granzyme B, T-bet and Eomes as well as IFN-γ, respectively. Vertical dotted lines indicate populations expressing the respective proteins based on the negative controls. **(B)** Diagrams showing the relative mean fluorescence intensity (MFI) of indicated proteins in the mutant CTLs (the values of WT cells set as 1) **(C)** Histograms showing CD8α expression in the mutant CTLs. Th2 cells were included as a negative control. Vertical dotted line indicates peak of CD8α expression in WT CTLs. **(D)** Diagrams showing the MFI of CD8α expression in the mutant CTLs (the values of WT cells set as 1). **(B, D)** For the comparison of values between three mutant groups, a one-way ANOVA analysis was performed. The *p*-values were defined as following: *p < 0.05; **p < 0.01; ***p < 0.001. For the comparison of the relative MFI values between WT (set as 1) and each mutant group, a one-sample *t*-test was performed. The *p*-values were indicated above the diagrams. Data are representative **(A, C)** or show the summary **(B, D)** of 4-6 independent experiments.

To identify the unique role for MAZR as well as its potential synergistic activity with Runx3 during CTL differentiation, we utilized previously generated mice with a T cell lineage-specific deletion of MAZR (*Mazr* ^f/f^ *Cd4*-Cre), Runx3 (*Runx3* ^f/f^ *Cd4*-Cre) as well as MAZR and Runx3 double-deficient (*Mazr* ^f/f^ *Runx3* ^f/f^ *Cd4*-Cre) mice (hereafter referred to as MAZR-cKO^CD4^, Runx3-cKO^CD4^ and M/R3-cDKO^CD4^ mice, respectively) (13). As previously reported (13, 21, 36), Runx3-cKO^CD4^ and M/R3-cDKO^CD4^ CD8^+^ T cells partially derepressed CD4 (**Figure S1A**), and the analysis of the CD44/CD62L expression pattern showed a slightly increased proportion of the CD44^hi^CD62L^–^ subset in the absence of Runx3, compared to wild-type control mice (*Mazr* ^f/f^ *Runx3* ^f/f^, hereafter referred to as WT mice) (**Figures S1A, B**). We isolated CD44^lo^CD62L^+^ naïve CD8^+^ T cells from the various mutants (including CD4^med-hi^CD8^+^ T cell population in Runx3-cKO^CD4^ and M/R3-cDKO^CD4^ mice) and stimulated them *in vitro* with anti-CD3 and anti-CD28 antibodies. Naïve CD8^+^ T cells have been shown to differentiate *in vitro* during a period of 6 days into fully armed effector CTLs as characterized by high expression levels of Granzyme B, T-bet, Eomes and IFN-γ (37). As previously reported (37), Runx3-cKO^CD4^ CTLs displayed a reduction in Granzyme B and a severe loss of Eomes expression during CTL differentiation (**Figures 1A, B**). While MAZR-deficient CTLs did not show altered expression of Granzyme B and Eomes, the deletion of both MAZR and Runx3 led to a further downregulation in Granzyme B expression in comparison to Runx3-cKO^CD4^ CTLs (**Figures 1A, B**). Moreover, whereas there was a mild reduction in T-bet expression in MAZR-cKO^CD4^ and also a tendency in Runx3-cKO^CD4^ CTLs, M/R3-cDKO^CD4^ CTLs displayed a greater degree of its downmodulation, indicating an essential role for the combined activity of the two factors (**Figures 1A, B**). To study effector function in the absence of MAZR and/or Runx3, we also examined cytokine production and cytotoxic activity of the mutant CTLs. While there was no alteration in the expression level of IFN-γ in the absence of MAZR, R3-cKO^CD4^ CTLs displayed a reduced level of IFN-γ expression, and a similar reduction was also observed in M/R3-cDKO^CD4^ CTLs (**Figures 1A, B**). There was also a similar tendency of change in IL-2 expression pattern, while TNF-α expression was not altered in the mutant cells (**Figures S1C, D**). Redirected cytotoxicity assay revealed that, while MAZR-cKO^CD4^ CTLs displayed a similar degree of cytotoxic activity to WT CTLs, the deletion of Runx3 led to almost complete abolishment of the activity (**Figure S1E**). Finally, as we have previously shown (38), loss of Runx3 led to a down-regulation of CD8α expression in comparison to WT cells (**Figures 1C, D**). While CD8α expression levels were also mildly affected by the loss of MAZR, the combined deletion of both MAZR and Runx3 led to a further downregulation of CD8α expression in comparison to the single mutant CTLs (**Figures 1C, D**). Of note, unlike Runx3-cKO^CD4^ CTLs, the deletion of MAZR (both on WT and Runx3-deficient backgrounds) had no impact on the proliferation and survival of activated CD8^+^ T cells (**Figures S2A–E**), indicating that the phenotypic alterations observed in MAZR-cKO^CD4^ and M/R3-cDKO^CD4^ CTLs (compared to WT and Runx3-cKO^CD4^ CTLs, respectively) were not due to defects in the expansion of CD8^+^ T cells. In addition, the impact of MAZR deletion was not yet evident at the early activation phase (i.e. day 3), suggesting that MAZR regulates CD8α, Granzyme B and T-bet expression at the later stage of CTL differentiation (**Figure S2F, G**). Together, these results indicate that, in contrast to an essential role of Runx3 for the expression of key CTL proteins and cytotoxic activity, the deletion of MAZR has a mild effect on CD8α and T-bet expression. However, on a Runx3-deficient background, additional loss of MAZR leads to a further reduction of Granzyme B expression, suggesting that MAZR partially compensates for loss of Runx3 in CTLs, albeit this appears to be not linked with cytotoxic activity. Moreover, the degree of reduction in CD8α and T-bet expression was greater in M/R3-cDKO^CD4^ CTLs compared to the single mutant cells, indicating synergistic activities of MAZR and Runx3 in CTLs in the regulation of the two molecules.

### Ectopic Expression of MAZR Partially Reverses the Defects in CTL Differentiation by Combined Loss of MAZR and Runx3

**Figure 2:**
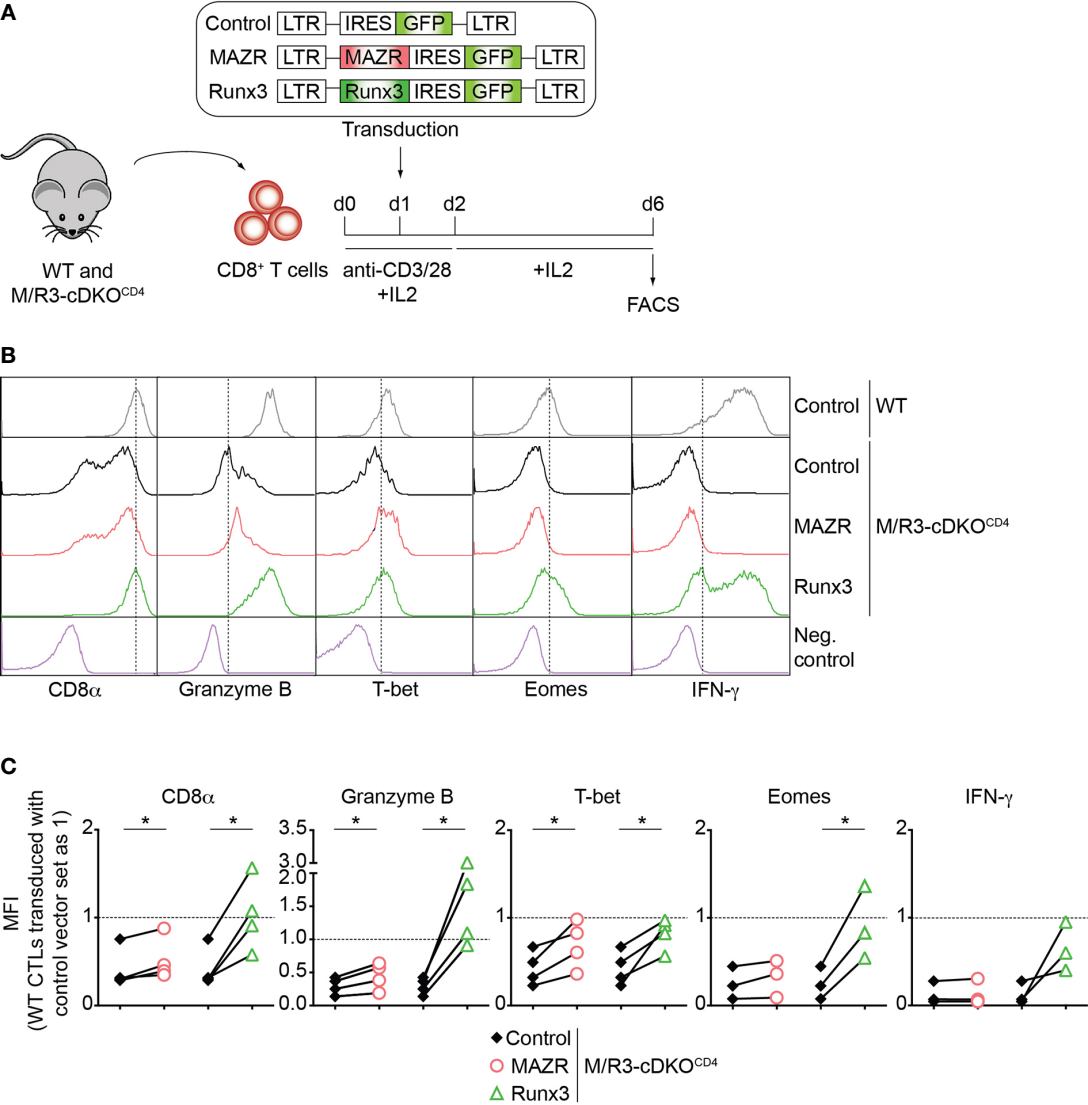
Enforced expression of MAZR partially restores impaired CTL differentiation in the absence of MAZR and Runx3. **(A)** Schematic figure showing the retroviral vector-mediated gene transduction of MAZR or Runx3 into WT and M/R3-cDKO^CD4^ CTLs. See the Methods section for the detailed experimental procedure. **(B)** Histograms showing CD8α, Granzyme B, T-bet, Eomes and IFN-γ expression in GFP^+^ WT CTLs transduced with a control vector as well as in GFP^+^ M/R3-cDKO^CD4^ CTLs transduced with control, MAZR- and Runx3-expressing vectors (6 days after *in vitro* anti-CD3/28 stimulation). T helper 2 (Th2) cells were included as negative controls for CD8α, Granzyme B, T-bet and Eomes stainings, whereas WT CTLs without restimulation were used as a negative control for IFN-γ staining. Vertical dotted lines indicate the peak of CD8α expression in WT CTLs or populations expressing the respective proteins based on the negative controls. **(C)** Diagrams showing the relative mean fluorescence intensity (MFI) of CD8α, Granzyme B, T-bet, Eomes and IFN-γ expression in GFP^+^ M/R3-cDKO^CD4^ CTLs transduced with control, MAZR- and Runx3-expressing vectors. The MFI values of GFP^+^ WT CTLs transduced with control vector were set as 1, and are indicated as horizontal dotted lines. Each dot represents value from each experiment, and paired experiments are indicated with lines. A paired two-tailed Student’s *t*-test was performed for statistical analysis. The *p*-values were defined as following: *p < 0.05. Data are representative **(B)** or show the summary **(C)** of 3-4 independent experiments.

To further study unique and/or combined activities of MAZR and Runx3 in CD8^+^ T cells, we next examined whether enforced MAZR or Runx3 expression reverts the impaired CTL differentiation phenotype observed in MAZR/Runx3-deficient CTLs (**Figure 2A**). While retroviral-mediated overexpression of Runx3 restored the expression of all factors analyzed to almost the levels observed in control vector (“empty”)-transduced WT CD8^+^ T cell, enforced MAZR expression partially compensated for the loss of Runx3 with respect to restoring the expression of CD8α, Granzyme B and T-bet (**Figures 2B, C**). Thus, these results underline a modulatory role of MAZR in the expression of a specific set of genes during CTL differentiation.

### Deletion of MAZR and Runx3 Causes Impaired CTL Differentiation in a ThPOK-Independent Manner

**Figure 3:**
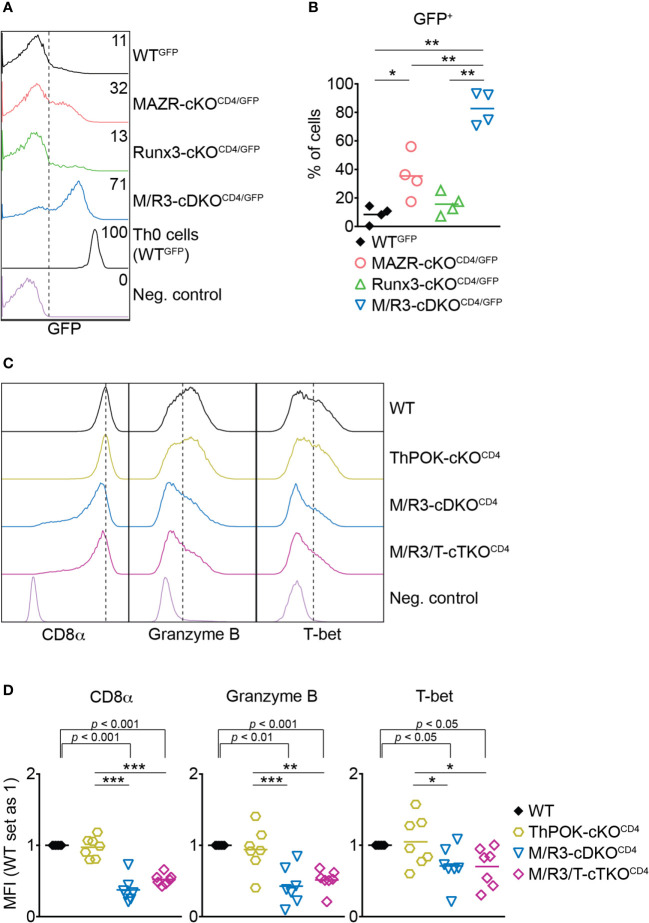
The combined deletion of MAZR and Runx3 leads to impaired CTL differentiation independently of enhanced ThPOK expression. **(A)** Histograms showing GFP expression in the mutant CTLs (6 days after *in vitro* anti-CD3/28 stimulation) as well as WT^GFP^ Th0 cells. WT CTLs were used as a negative control for the analysis of GFP expression. **(B)** Diagrams showing the percentages of GFP^+^ population in the mutant CTLs. **(C)** Histograms showing the expression of indicated proteins in the mutant CTLs (6 days after *in vitro* anti-CD3/28 stimulation). Vertical dotted lines indicate the peak of CD8α expression in WT CTLs or populations expressing Granzyme B and T-bet based on the negative controls. **(D)** Diagrams displaying the relative mean fluorescence intensity (MFI) of indicated proteins in the mutant CTLs (the values of WT cells set as 1). For the comparison of relative MFI values between WT (set as 1) and each mutant group, a one-sample *t*-test was performed. The *p*-values were indicated above the diagrams. **(B,D)** For the comparison of values between four groups **(B)** and three mutant groups **(D)**, a one-way ANOVA analysis was performed. The *p*-values were defined as following: *p < 0.05; **p < 0.01; ***p < 0.001. **(D)** Data are representative **(A, C)** or show the summary **(B, D)** of 4 **(A, B)** and 7 **(C, D)** mice analyzed in 4 **(A, B)** and 5 **(C, D)** independent experiments.

ThPOK is a central transcription factor for helper lineage differentiation and required for the maintenance of the lineage integrity (39). We previously demonstrated that MAZR and Runx3 synergistically repress ThPOK during CD8^+^ T cell development and that the combined deletion of MAZR and Runx3 led to the derepression of ThPOK in approximately 30% of peripheral CD8^+^ T cells (13). We therefore examined whether the cooperative activity of those two factors is also required for ThPOK repression in CTLs. To easily monitor *Thpok* expression in mutant CTLs, we took advantage of previously generated MAZR-cKO^CD4^, Runx3-cKO^CD4^ and M/R3-cDKO^CD4^ mice that were crossed with a ThPOK-GFP knock-in reporter strain (referred to as MAZR-cKO^CD4/GFP^, Runx3-cKO^CD4/GFP^ and M/R3-cDKO^CD4/GFP^, respectively) (13). In line with previous reports (13, 40) a fraction of WT^GFP^ CTLs expressed GFP (i.e. *Thpok*), while MAZR-cKO^CD4/GFP^ CTLs displayed an increased proportion of GFP^+^ CTLs (**Figures 3A, B**). Notably, whereas Runx3-cKO^CD4/GFP^ CTLs express GFP at a similar level as WT cells, the combined loss of MAZR and Runx3 resulted in the derepression of ThPOK in a majority (approx. 80%) of CTLs, although their GFP expression level was lower compared to the one in the helper lineage (**Figures 3A, B**). These data demonstrate that combined activity of MAZR and Runx3 is also required for ThPOK repression in CTLs.

A previous study showed that ectopic expression of ThPOK in CTLs leads to a reduction in the expression of CD8 and cytotoxic effector genes including Granzyme B (41). It is therefore possible that the impaired expression of effector genes in MAZR/Runx3-deficient CTLs is a consequence of increased ThPOK expression. In order to test this possibility, we crossed M/R3-cDKO^CD4^ mice with mice having a conditional *Thpok* allele (22) to generate MAZR/Runx3/ThPOK triple-deficient mice (referred to as M/R3/T-cTKO^CD4^). M/R3/T-cTKO^CD4^ CTLs displayed similar degrees of reduction in CD8α, Granzyme B and T-bet expression as observed in M/R3-cDKO^CD4^ cells (**Figures 3C, D**), indicating that the impaired expression of these molecules in MAZR/Runx3-deficient CTLs is not due to the enhanced expression of ThPOK.

### MAZR Plays a Compensatory Role in the Runx3-Dependent Transcriptional Program of CTL Differentiation

**Figure 4:**
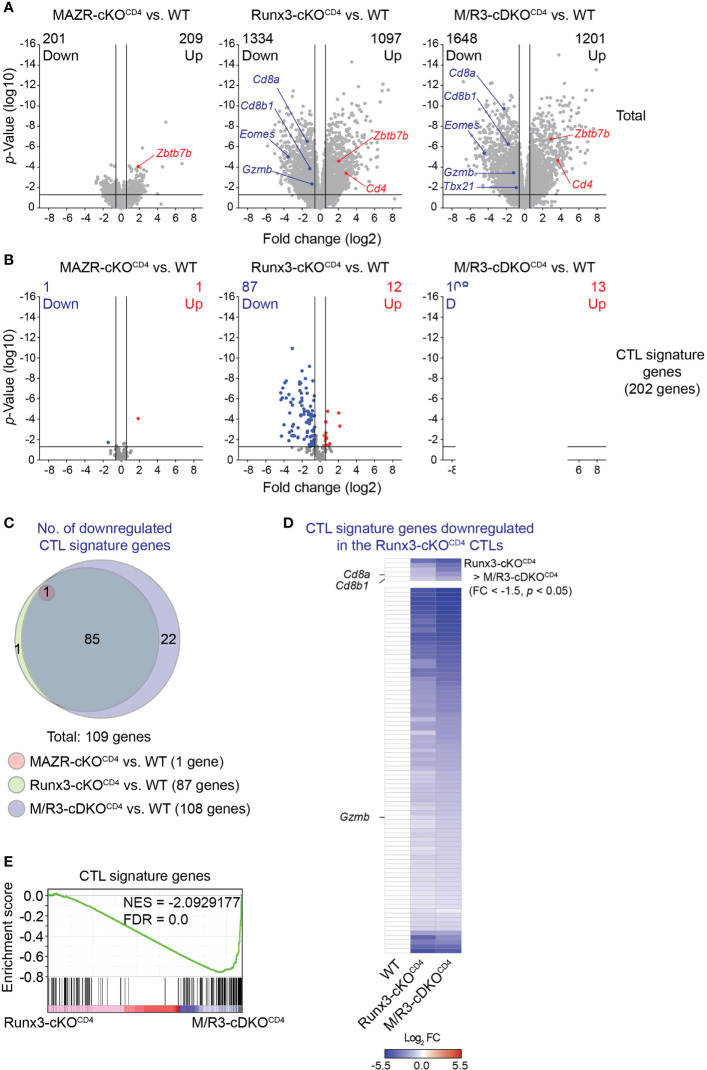
MAZR plays a compensatory role in the Runx3-dependent transcriptional program of CTL differentiation. **(A)** Volcano plots showing differentially expressed genes in the individual mutant CTLs compared to WT cells (6 days after *in vitro* anti-CD3/28 stimulation). **(B)** Volcano plots showing the expression pattern of the 202 CTL signature genes in the individual mutant CTLs compared to WT cells. **(C)** Venn diagrams displaying the overlaps of the downregulated CTL signature genes in the individual mutant CTLs compared to WT cells. Numbers of gene in the respective regions are indicated. **(D)** Heatmap showing fold change (FC; log2 transformed) differences of 87 CTL signature genes downregulated in R3-cDKO^CD4^ CTLs compared to WT cells. The expression patterns of the genes in R3-cDKO^CD4^ and M/R3-cDKO^CD4^ CTLs are shown (mean expression levels of WT CTLs were set as 1). Five genes further downmodulated in M/R3-cDKO^CD4^ CTLs (based on a threshold of FC > 1.5 and *p*-value < 0.05) and the rest 82 genes are presented in the upper and lower panels, respectively. Names of the selected genes (i.e. the *Cd8a*, *Cd8b1* and *Gzmb* genes) are indicated at the left. **(E)** Gene set enrichment analysis of the CTL signature genes in the gene expression profile of M/R3-cDKO^CD4^ CTLs in comparison to Runx3-cDKO^CD4^. NES, normalized enrichment score; FDR, false-discovery rate. **(A,B)** Cutoff lines of 1.5-fold change as well as *p*-value < 0.05 were indicated in plots.

In order to gain insight into the transcriptional program controlled by MAZR and Runx3 in CTLs, we performed RNA sequencing (RNA-seq) experiments and compared genome-wide transcriptional profiles of *in vitro*-activated WT, MAZR-cKO^CD4^, Runx3-cKO^CD4^ and M/R3-cDKO^CD4^ CTLs. Overall, the deletion of Runx3 had a greater impact on gene expression in CTLs (2431 genes dysregulated) in comparison to MAZR-deficient CTLs (410 genes dysregulated) (**Figure 4A**). However, the combined deletion of MAZR and Runx3 resulted in a more severe alteration in the transcriptome (2849 genes dysregulated) than Runx3-deficient CTLs (**Figure 4A**). In line with the results obtained from the flow cytometric analysis (**Figure 1**), the RNA-seq of M/R3-cDKO^CD4^ CTLs confirmed the downregulation of *Cd8a*, *Cd8b1*, *Eomes*, *Gzmb* (encoding Granzyme B) and *Tbx21* (encoding T-bet) as well as the upregulation of *Zbtb7b* (encoding ThPOK), and some of the gene expression patterns were further validated by quantitative real-time PCR (**Figure S3A**). This indicates that the expression of these factors is controlled at the transcriptional level by MAZR and Runx3. The comparison of differentially expressed genes in the individual mutant CTLs showed that there was a substantial overlap between Runx3- and MAZR/Runx3-depenedent genes (**Figures S3B, C**). Nonetheless, a set of genes were uniquely dysregulated in MAZR-deficient CTLs, suggesting that MAZR regulates distinct pathways/biological processes in CTLs. To further address the impact of MAZR and Runx3 on transcriptional programs during CTL differentiation, we investigated the expression pattern of CTL signature genes in WT, MAZR-cKO^CD4^, Runx3-cKO^CD4^ and M/R3-cDKO^CD4^ CTLs. Using the Immunological Genome Project (ImmGen) database (33), we determined the top 200 genes that are highly expressed in CTLs 8 days after vesicular stomatitis Indiana virus (VSV) infection in comparison to naïve CD8^+^ T cells (34). By adding *Cd8a* and *Cd8b1* genes we defined a CTL signature gene set containing 202 genes. While loss of MAZR led to minor changes in the expression pattern of these CTL signature genes (one gene was up- and downregulated, respectively), Runx3-cKO^CD4^ CTLs up- and downregulated 12 and 87 CTL signature genes, respectively, in comparison to WT CTLs (**Figure 4B**), thus confirming the key role of Runx3 for CTL differentiation (17, 18, 37, 42). However, the combined deletion of MAZR and Runx3 led to a further increase in the number of downregulated CTL signature genes (i.e. from 87 to 108 genes) (**Figure 4B**) due to reduced expression of an additional set of CTL signature genes (**Figure 4C**). Moreover, among 87 signature genes downregulated in Runx3-cKO^CD4^ CTLs, five genes showed further downmodulation in M/R3-cDKO^CD4^ cells (based on a threshold of fold change > 1.5 and *p*-value < 0.05, **Figure 4D** upper panel), and 72 additional genes displayed a greater degree of reduction in their expression levels upon the combined deletion, albeit the differences did not reach the aforementioned threshold (**Figure 4D** lower panel). These results indicate that M/R3-cDKO^CD4^ CTLs display a more severe impairment of CTL signature gene expression in comparison to Runx3-cKO^CD4^ CTLs. Of note, the analysis of upregulated CTL signature genes showed that the additional deletion of MAZR on a Runx3-deficient background had a lesser impact on a change in gene expression (**Figures S3D, E**). Finally, consistent with the further downregulation of CTL signature genes by the combined deletion of MAZR and Runx3, gene set enrichment analysis (GSEA) revealed that CTL signature genes were underrepresented in M/R3-cDKO^CD4^ CTLs in comparison to Runx3-cKO^CD4^ cells (**Figure 4E**). Together, the RNA-seq analysis underscores a compensatory role of MAZR for Runx3-dependent transcriptome changes during CTL differentiation.

### Altered *In Vitro* CTL Differentiation by Loss of MAZR and/or Runx3 Is Largely Due to CD8^+^ T Cell Lineage-Intrinsic Defects

**Figure 5:**
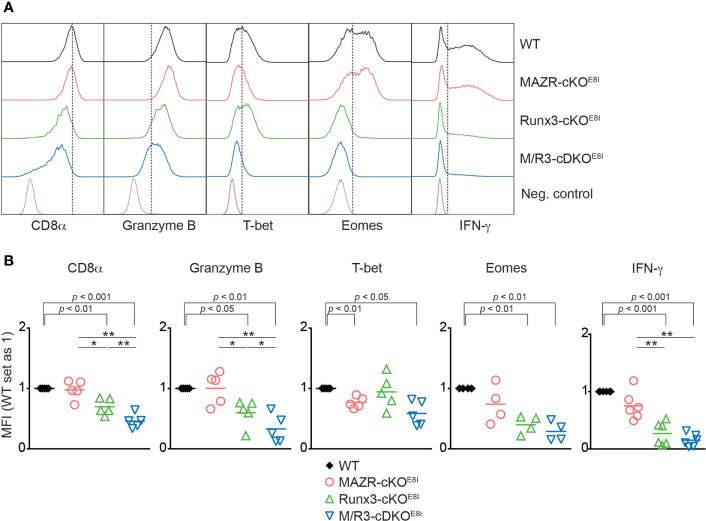
Altered *in vitro* CTL differentiation by loss of MAZR and/or Runx3 is largely due to CD8^+^ T cell lineage-intrinsic defects. **(A)** Histograms showing the expression of indicated proteins in the mutant CTLs (6 days after *in vitro* anti-CD3/28 stimulation). T helper 2 (Th2) cells as well as WT CTLs without restimulation were included as negative controls for the stainings of Granzyme B, T-bet and Eomes as well as IFN-γ, respectively. Vertical dotted lines indicate the peak of CD8α expression in WT CTLs or populations expressing the respective proteins based on the negative controls. Due to the appearance of Runx3-expressing cells within Runx3-cKO^E8I^ and M/R3-cDKO^E8I^ CTLs (that had escaped from Cre-mediated excision of the *Runx3* allele), the expression pattern in the two mutant cells were analyzed in the Runx3-negative population by including intracellular staining of Runx3 protein. **(B)** Diagrams showing the relative mean fluorescence intensity (MFI) of indicated proteins in the mutant CTLs (the values of WT cells set as 1). For the comparison of values between three mutant groups, a one-way ANOVA analysis was performed. The *p*-values were defined as following: *p < 0.05; **p < 0.01. For the comparison of the relative MFI values between WT (set as 1) and individual mutant CTLs, a one-sample *t*-test was performed. The *p*-values were indicated above the diagrams. Data are representative **(A)** or show the summary **(B)** of 4-6 mice analyzed in 4-6 independent experiments.

We have previously demonstrated that the combined deletion of MAZR and Runx3 during thymocyte development (using the *Cd4*-Cre strain) leads to the derepression of helper lineage genes in peripheral CD8^+^ T cells (13). Therefore, the phenotypic alterations observed in the mutant CTLs on a *Cd4*-Cre background might be, at least in part, due to defects in CD8 lineage differentiation during T cell development. In order to address this issue, we utilized an *E8I*-Cre strain to delete MAZR and/or Runx3, where the Cre recombinase is specifically active in mature CD8 lineage T cells (i.e. in mature CD8 SP thymocytes and peripheral CD8^+^ T cells) (26). This led to the generation of *Mazr* ^f/f^ *E8I*-Cre, *Runx3* ^f/f^ *E8I*-Cre, *Mazr* ^f/f^ *Runx3* ^f/f^ *E8I*-Cre mice (hereafter referred to as MAZR-cKO^E8I^, Runx3-cKO^E8I^ and M/R3-cDKO^E8I^ mice, respectively). We differentiated the mutant CD8^+^ T cells on an *E8I*-Cre background *in vitro* and examined the expression patterns of the key CTL proteins. This analysis showed that the deletion of MAZR and/or Runx3 by *E8I*-Cre led to similar phenotypic alterations (**Figures 5A, B**) as observed in the mutant cells on a *Cd4*-Cre background (**Figure 1**). However, unlike MAZR-cKO^CD4^ CTLs, MAZR-cKO^E8I^ CTLs displayed no alteration in CD8α expression and a tendency of reduction in Eomes and IFN-γ expression levels. In addition, there was a greater degree of impairment in T-bet expression upon *E8I*-Cre-mediated MAZR deletion. Of note, a fraction of cells “escaped” Cre-mediated deletion of Runx3 in Runx3-cKO^E8I^ and M/R3-cDKO^E8I^ CTLs, and the expression pattern of key CTL proteins was therefore assessed within the Runx3-negative population (as determined by intracellular Runx3 staining). These data indicate that altered expression of CTL-characteristic proteins by loss of MAZR and/or Runx3 is largely due to CD8 lineage-intrinsic defects.

### MAZR and Runx3 Cooperatively Regulate CTL Differentiation in Response to Viral Infection

**Figure 6:**
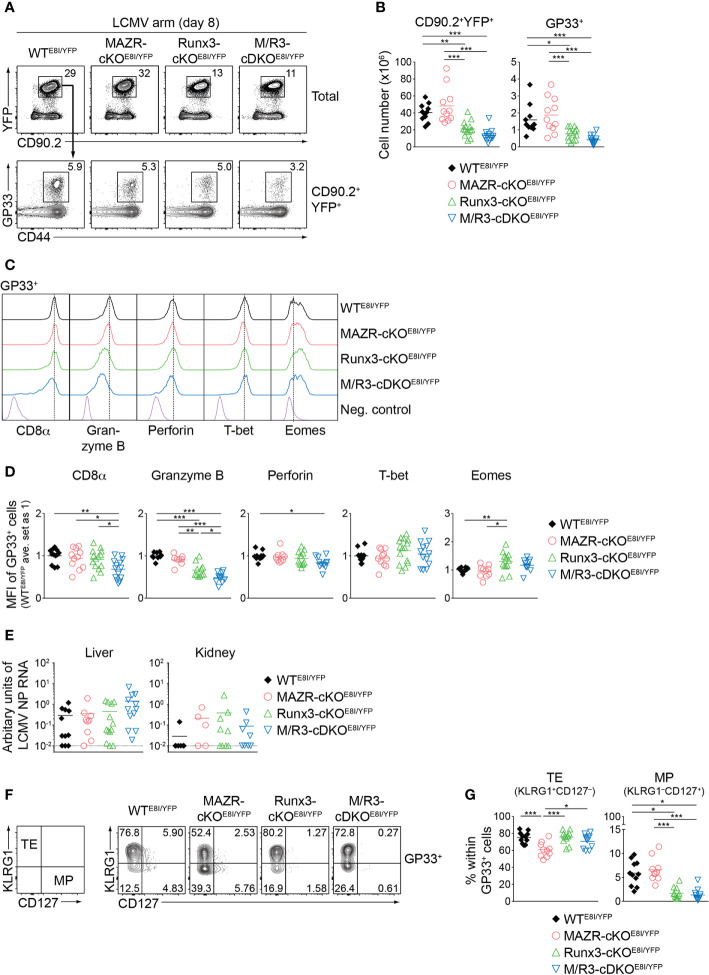
MAZR and Runx3 cooperatively regulate *in vivo* CTL differentiation in response to viral infection. **(A)** Flow cytometric analysis showing CD90.2 and YFP expression in total splenocytes (upper panel), CD44 expression and H2-D^b^/GP33-41 tetramer (GP33) staining (lower panel) of CD90.2^+^YFP^+^ splenocytes of the mutant mice (8 days after LCMV Armstrong infection). **(B)** Diagrams showing the cell numbers of CD90.2^+^YFP^+^, and CD90.2^+^YFP^+^GP33^+^ (GP33^+^) splenocytes of the mutant mice. **(C)** Histograms showing the expression of indicated proteins in GP33^+^ splenocytes of the indicated genotypes. As negative staining controls naïve CD4^+^ T cells (for CD8α) and naïve CD8^+^CD44^lo^ cells (for Granzyme B, Perforin, T-bet and Eomes) from uninfected WT mice were included. Vertical dotted lines indicate the peaks of expression of the individual proteins in WT CTLs. **(D)** Diagrams showing the relative mean fluorescence intensity (MFI) of indicated proteins in GP33^+^ splenocytes of the indicated genotypes. The average values of WT^E8I/YFP^ mice were set as 1. **(E)** qRT-PCR analysis of the abundance of transcripts encoding LCMV nucleoprotein (NP) in the livers and kidneys isolated from mice of the indicated genotypes. Results are presented relative to those of control transcripts encoding ribosomal protein lateral stalk subunit P0 (*Rplp0*). The long dashed horizontal lines indicate the limit of detection. **(F)** Flow cytometric analysis showing KLRG1 and CD127 expression in GP33^+^ splenocytes of the indicated. The panel on the left indicates the definition of individual subsets: KLRG1^+^CD127^–^ terminal effector (TE) and KLRG1^–^CD127^+^ memory precursor (MP) cells. **(G)** Diagrams show the percentage of MP and TE subsets within GP33^+^ splenocytes of the indicated genotypes. **(A,F)** Numbers indicate percentages of the respective subsets. **(B, D, E, G)** Each dot represents one mouse. The horizontal bars indicate the mean. *p < 0.05; **p < 0.01; ***, *p* < 0.001 (one-way ANOVA analysis followed by Tukey’s multiple-comparison test). Data are representative **(A, C, F)** or show the summary **(B, D, E, G)** of more than 10 mice analyzed in at least 5 independent experiments (except for the analysis of viral titers in kidney **(E)**, where 5-9 mice from 6 independent experiments were included).

We next examined the impact of MAZR- and/or Runx3-deficiency on CTL differentiation *in vivo* upon acute viral infection. For that, we utilized the mutant mice on an *E8I*-Cre background and further introduced a *Rosa26*-EYFP reporter allele to monitor Cre activity. This led to the generation of *Mazr* ^f/f^ *Rosa26^YFP/+^E8I*-Cre, *Runx3* ^f/f^ *Rosa26^YFP/+^E8I*-Cre, *Mazr* ^f/f^ *Runx3* ^f/f^ *Rosa26^YFP/+^E8I*-Cre and control *Rosa26^YFP/+^E8I*-Cre mice (hereafter referred to as MAZR-cKO^E8I/YFP^, Runx3-cKO^E8I/YFP^, M/R3-cDKO^E8I/YFP^ and WT^E8I/YFP^ mice, respectively). In agreement with previous studies (26, 43), virtually all YFP^+^ peripheral T cells in WT^E8I/YFP^ mice were CD8^+^ T cells (**Figure S4A**) and the efficient deletion of *Mazr* and/or *Runx3* in the mutant YFP^+^ T cells was confirmed by intracellular staining (**Figure S4B**). *E8I*-Cre-mediated deletion of MAZR and/or Runx3 did not lead to alterations in the number as well as the homeostatic status (based on CD44/CD62L expression) of YFP^+^ cells (**Figure S4A, C**) and, unlike in Runx3-deficient mice on a *Cd4*-Cre background, no apparent CD4 derepression was observed in Runx3-cKO^E8I/YFP^ and M/R3-cDKO^E8I/YFP^ YFP^+^ T cells (**Figure S4A**). We infected the mutant mice on a *E8I*-Cre/*Rosa26*-EYFP background with the LCMV Armstrong strain and performed immunophenotyping of virus-specific CD8^+^ T cells eight days after infection (44). In MAZR-cKO^E8I/YFP^ mice, there was no alteration in the numbers of total YFP^+^ T cells as well as glycoprotein 33-41 (GP33)-specific CTLs, compared to WT^E8I/YFP^ mice (**Figures 6A, B**). In contrast, Runx3-cKO^E8I/YFP^ mice displayed reduced numbers of total YFP^+^ T cells and GP33-specific CTLs, similar to the observations made in previous studies (17, 18) (**Figures 6A, B**). However, M/R3-cDKO^E8I/YFP^ mice showed a tendency of a further reduction in their cell numbers in comparison to Runx3-cKO^E8I/YFP^ mice (**Figures 6A, B**), and a similar tendency was also observed for the number of nucleoprotein 396-404 (NP396)-specific CTLs (**Figures S5A, B**). These results suggest the modulatory role of MAZR for clonal expansion of CTLs, at least in the absence of Runx3.

We then characterized CD8α, Granzyme B, perforin, T-bet and Eomes expression in the mutant CTLs. This analysis revealed that the combined deletion of MAZR and Runx3 led to downmodulation of CD8α expression in comparison to WT and the individual deletions, in both GP33- (**Figures 6C, D**) and NP396- (**Figures S5C, D**) specific CTLs. In addition, Runx3-cKO^E8I/YFP^ CTLs expressed Granzyme B at a lower level than WT^E8I/YFP^ CTLs, and the combined loss of MAZR and Runx3 led to a further reduction of Granzyme B expression. Moreover, the expression level of perforin was lower in M/R3-cDKO^E8I/YFP^ CTLs in comparison to WT^E8I/YFP^ cells, despite its comparison to single mutant CTLs did not reach a statistically significant level. These results suggest that MAZR and Runx3 cooperatively regulate CD8α and perforin expression, and that their combined activity is required for appropriate expression of Granzyme B. In contrast, there was no alteration observed in the regulation of T-bet and Eomes in M/R3-cDKO^E8I/YFP^ CTLs, whereas Runx3-cKO^E8I/YFP^ CTLs showed an increase in Eomes expression compared to WT cells (**Figures 6C, D**). In order to assess effector function of the mutant CTLs we examined the surface expression of CD107 protein, which is a marker for cytotoxic degranulation (45), as well as cytokine production upon GP33 peptide restimulation. This analysis revealed that, while Runx3-cKO^E8I/YFP^ CTLs displayed an alteration in cytokine expression pattern as well as a tendency of reduced CD107 expression, the deletion of MAZR alone as well as the additional deletion of MAZR on a Runx3-deficient background had no impact on their expression (**Figures S5E, F**). However, there was a tendency that viral loads in the mutant mice were increased in the liver of M/R3-cDKO^E8I/YFP^ mice, compared to WT^E8I/YFP^ and single mutant mice (**Figure 6E**). This suggests that MAZR and Runx3 cooperatively contribute to viral control, possibly in part *via* regulating the expansion of CTLs and/or the expression of cytolytic proteins.

Finally, since a previous study demonstrated an essential role of Runx3 for promoting memory cell formation (18, 46), we investigated the impact of MAZR- and/or Runx3-deletion on the generation of memory precursor (MP) CTLs. For this, we examined KLRG1 and CD127 expression patterns of the mutant CTLs, which allows us to identify KLRG1^–^CD127^+^ MP and KLRG1^+^CD127^–^ terminal effector (TE) CTLs (5, 8, 10). Consistent with the previous report (18), GP33-specific Runx3-cKO^E8I/YFP^ CTLs displayed impaired MP cell differentiation, and the additional deletion of MAZR led to a similar degree of the impairment (**Figures 6F, G**). In contrast, MAZR-cKO^E8I/YFP^ CTLs showed a reduction in the proportion of TE cells, while there was no change in MP cells (**Figures 6F, G**). NP396-specific mutant CTLs displayed similar alterations in KLRG1 and CD127 expression patterns (**Figures S5G, H**). These results indicate that MAZR and Runx3 play different roles for TE-versus-MP subset differentiation, albeit impaired TE cell differentiation by loss of MAZR become less evident on a Runx3-deficient background. In summary, the phenotypic analyses of virus-specific CTLs revealed that a combined activity of MAZR and Runx3 is required for the acquisition of certain CTL characteristics *in vivo*. In addition, they suggest distinct roles of the two molecules for the generation of memory CD8^+^ T cell precursor.

### MAZR and Runx3 Exert Distinct Functions for the Generation of Memory T Cell Subsets

**Figure 7:**
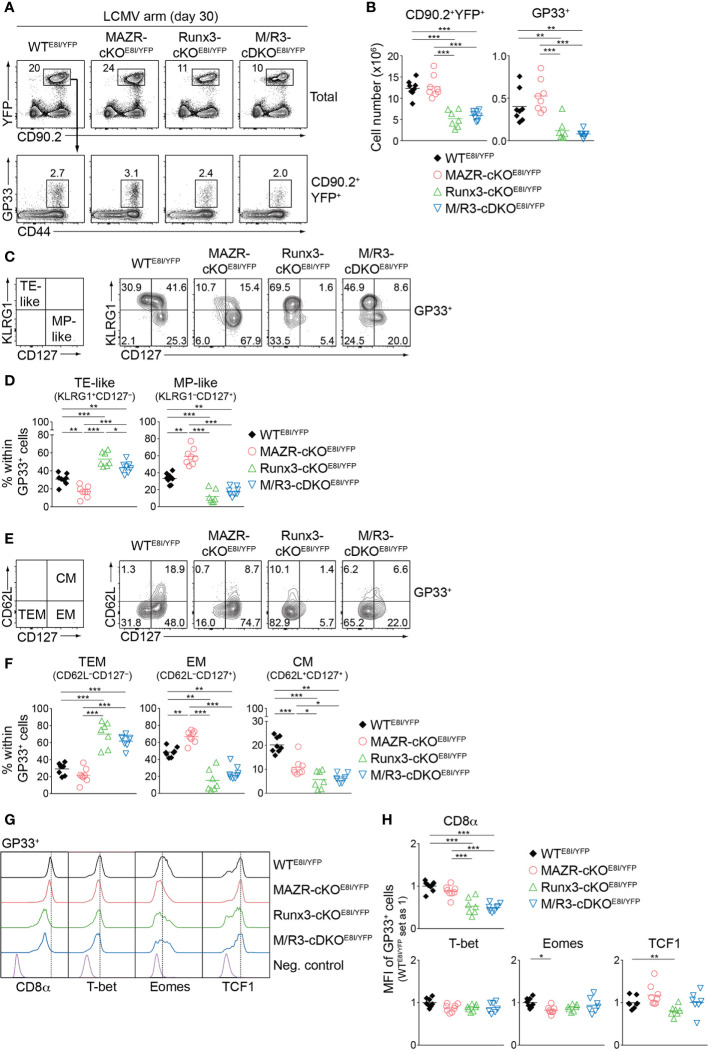
MAZR and Runx3 exerts distinct function for the generation of memory T cell subsets. **(A)** Flow cytometric analysis showing CD90.2 and YFP expression in total splenocytes (upper panel) and CD44 expression and H2-D^b^/GP33-41 tetramer (GP33) staining of CD90.2^+^YFP^+^ splenocytes (lower panel) isolated from mice of the indicated genotypes (thirty days after LCMV Armstrong infection). **(B)** Diagrams showing the cell numbers of CD90.2^+^YFP^+^ and CD90.2^+^YFP^+^GP33^+^ (GP33^+^) splenocytes of the indicated genotypes. **(C)** Flow cytometric analysis showing KLRG1 and CD127 expression in GP33^+^ splenocytes of the indicated genotypes. The panel on the left indicates the definition of individual subsets: KLRG1^+^CD127^–^ terminal effector-like (TE-like) and KLRG1^–^CD127^+^ memory precursor-like (MP-like) cells. **(D)** Diagrams showing the percentage of the indicated subsets [identified as shown in **(C)**] within GP33^+^ splenocytes of the indicated genotypes. **(E)** Flow cytometric analysis showing CD62L and CD127 expression in GP33^+^ splenocytes of the indicated genotypes. The panel on the left indicates the definition of individual subsets: CD62L^–^CD127^+^ terminal effector memory (TEM), CD62L^–^CD127^+^ effector memory (EM) cells and CD62L^+^CD127^+^ central memory (CM) effector. **(F)** Diagrams showing the percentage of the indicated subsets (identified as shown in (**E**)) within GP33^+^ splenocytes of the indicated genotypes. **(G)** Histograms showing the expression of indicated proteins in GP33^+^ splenocytes of the indicated genotypes. As negative staining controls naïve CD4^+^ T cells (for CD8α), naïve CD8^+^CD44^lo^ cells (for T-bet and Eomes) and CD19^+^ B cells (for TCF1) from uninfected WT mice were included. **(H)** Diagrams showing the relative mean fluorescence intensity (MFI) of indicated proteins in GP33^+^ splenocytes of the indicated genotypes. The average values of WT^E8I/YFP^ mice were set as 1. **(A, C, E)** Numbers indicate percentages of the respective subsets. **(B, D, F, H)** Each dot represents one mouse. The horizontal bars indicate the mean. *p < 0.05; **p < 0.01; ***p < 0.001 (one-way ANOVA analysis followed by Tukey’s multiple-comparison test). Data are representative **(A, C, E)** or show the summary **(B, D, F, H)** of 7-8 mice analyzed in 2 independent experiments.

Having demonstrated altered TE/MP subset distribution in MAZR- and/or Runx3-deficient CTLs, we next characterized memory T cells in the mutant mice thirty days after infection. In line with a previous report (18), Runx3-cKO^E8I/YFP^ mice showed reduced numbers of CD90.2^+^YFP^+^ T cells, and the deletion of MAZR had no impact on their numbers both in WT and Runx3-deficient backgrounds (**Figures 7A, B**). Similar patterns of changes in cell numbers were also observed for the mutant memory T cells specific for GP33 (**Figures 7A, B**) and NP396 (**Figures S6A, B**). We then assessed the distribution of KLRG1^–^CD127^+^ and KLRG1^+^CD127^–^ subsets in virus-specific memory T cells [hereafter designated as MP- and TE-like subsets, respectively, due to less clear relationship of the canonical markers to cell fate at memory time points (5, 47)]. Notably, this analysis revealed that MAZR-cKO^E8I/YFP^ GP33-specific T cells displayed an increase in the proportion of MP-like cells, along with reduced proportion of TE-like subset (**Figures 7C, D**). Moreover, while loss of Runx3 led to an increased percentage of TE-like subset (accompanied with an appearance of atypical KLRG1^–^CD127^–^ subset), additional deletion of MAZR slightly reverted the phenotype (**Figures 7C, D**). The mutant NP396-specific memory cells displayed similar alterations in the subset distribution (**Figures S6C, D**), and these results therefore indicate opposite function of MAZR and Runx3 for TE-like subset differentiation.

We further characterized the mutant memory T cells based on CD62L/CD127 expression pattern [introduced by a recent study from Goldrath’s lab (48)], which allows us to identify three memory T cell subsets: CD62L^–^CD127^–^ terminal effector memory [TEM, also known as long-lived effector CD8^+^ T cells (LLEC) (49)], CD62L^–^CD127^+^ effector-memory (EM) and CD62L^+^CD127^+^ central-memory (CM) subsets. While TEM cells display characteristics similar to TE cells (with a certain degree of longevity), EM and CM cells persist for extended periods of time (particularly CM cells) and preferentially localize in the vasculature and lymphoid tissues, respectively (4, 6, 7, 48, 49). The analysis of CD62L and CD127 expression revealed that the deletion of MAZR results in the enlargement of EM subset, along with a reduced proportion of CM cells (**Figures 7E, F**). In addition, while in Runx3-cKO^E8I/YFP^ mice there was almost complete loss of EM and CM subsets, the additional deletion of MAZR led to a tendency of increase in the proportion of EM subset (**Figures 7E, F**). The expression pattern of CD8α as well as key transcription factors largely reflected the altered subset distribution in the mutant mice (**Figures 7G, H**). The deletion of MAZR led to the downmodulation of Eomes, whose expression is lower in EM cells in comparison to CM cells (48). Moreover, CD8α and TCF1 expression is reduced in Runx3-cKO^E8I/YFP^ cells, which is in line with their lowest expression in TEM subset (48). Together, these results highlight a unique role of MAZR to restrain the generation of EM subset (possibly in part through regulating EM-versus-CM diversification processes), which is distinct from the function of Runx3 to promote long-lived memory T cell differentiation.

## Discussion

The transcription factors MAZR and Runx3 play an essential role for CD8^+^ T cell development, and synergistically repress ThPOK expression during the process (13). In the present study we examined the unique role of MAZR as well as its potential synergistic activity with Runx3 during CTL and memory T cell differentiation. Consistent with previous reports (17, 18, 37), our analysis of key CTL protein expression as well as genome-wide profiling of gene expression showed an essential role of Runx3 for CTL effector function. In contrast, the deletion of MAZR had a milder effect on expression patterns, indicating its less contribution to CTL differentiation. However, the additional deletion of MAZR on Runx3-deficient background results in much more widespread downregulation of CTL signature genes compared to single Runx3 deletion, indicating a compensatory role of MAZR for Runx3-mediated transcriptional program in CTLs. Interestingly, we observed that not all the Runx3-dependedent genes/proteins were further downmodulated upon the combined loss of MAZR and Runx3, suggesting that MAZR only partially compensates for loss of Runx3 in the regulation of a specific set of genes. This was further supported by our observation that ectopic expression of MAZR in M/R-cDKO^CD4^ CTLs restored the expression levels of some factors (i.e. CD8α, Granzyme B and T-bet) but not others (i.e. Eomes and IFN-γ). Our results also revealed that MAZR regulates a set of key CTL proteins in synergy with Runx3 [e.g. CD8α expression *in vitro* CTLs (**Figure 1**)], where the combined deletion of the two molecules resulted in a greater than additive reduction in their expression levels, compared to the individual deletions (see also **Figure S7A** for the definition of synergistic regulation). In line with this observation, our RNA-seq analysis showed that 21 out of 104 genes downregulated in all the three mutant CTLs (**Figure S3C**) are synergistically regulated by MAZR and Runx3 (**Figure S7B**). Moreover, since part of the 21 genes displayed subtle “additive” changes upon the combined deletion, MAZR and Runx3 might regulate some common target genes in a parallel manner (**Figure S7A**). Together, our data suggest that MAZR contributes to CTL differentiation *via* exerting a compensatory function for Runx3-mediated CTL programs. In addition, it regulates a smaller number of genes in a cooperative manner with Runx3, highlighting a complex interplay between the two molecules during the generation of CTLs.

Mechanisms by which MAZR compensates for loss of Runx3 in the regulation of a set of genes in CTLs remain to be elucidated. However, considering that MAZR represses ThPOK expression in immature thymocytes *via* interacting with Runx1 (13), a similar interaction might mediate Runx3-independent CTL gene regulation. Interestingly, our transcriptome data as well as immunoblotting analysis showed that the deletion of Runx3 led to the elevated expression of Runx1 (but not MAZR) (**Figures S7C, D** and data not shown). Hence, the enhanced activity of the MAZR/Runx1 complex (incl. their increased recruitment to the CTL gene loci) might be responsible for the compensatory function of MAZR. In order to test these hypotheses, it is essential to first identify genomic regions bound by MAZR (and Runx1) in CTLs on Runx3-sufficient and deficient backgrounds. Unfortunately, currently available anti-MAZR/PATZ1 antibodies are not suitable for ChIP-qPCR or ChIP-seq approaches in our hands (data not shown), and alternative approaches such as generating ChIP-grade anti-MAZR antibodies or *in vivo* tagging of MAZR have to be considered for future studies.

We demonstrated that the mutant CTLs on an *E8I*-Cre background displayed a similar expression pattern of key CTL proteins as observed upon *Cd4-*Cre-mediated deletion. However, there was also a slight difference in the pattern, such as no reduction in CD8α expression as well as a greater reduction in T-bet expression by loss of MAZR on the *E8I*-Cre background. Therefore, while the phenotypic changes upon deletion by *Cd4-*Cre are largely due to CD8^+^ T cell-intrinsic defects, to a certain degree they might result from altered thymic development in the mutant mice. Since we also used the *Cd4*-Cre deleter line to perform our transcriptome analysis, a fraction of differentially expressed genes might be regulated by MAZR and/or Runx3 during earlier developmental processes. Moreover, while MAZR has been shown to repress ThPOK expression in a post-thymic manner (13), for the rigorous assessment of MAZR/Runx3-mediated ThPOK repression and its impact on CTL function, compound mutant mice on an *E8I*-Cre (or an inducible Cre) background remains to be elucidated.

Our data indicate that a combined activity of MAZR and Runx3 is required for appropriate CD8 and Granzyme B expression during *in vitro* and *in vivo* CTL differentiation. In contrast, altered T-bet expression in the absence of both MAZR and Runx3 was only observed in *in vitro* but not in *in vivo* CTLs. Such phenotypic differences between *in vitro* and *in vivo* CTLs was also detected upon Runx3 deletion (e.g. Eomes expression), which is in line with the observation made in a previous study (17). Given that *in vivo* CTL differentiation is initiated by multiple and distinct factors such as TCR signal strength, inflammatory cytokines and tissue microenvironment (3, 50), it is conceivable that *in vitro* CTL generation does not fully follow the CTL differentiation process upon viral infection. Indeed, recent ATAC-seq analysis revealed a substantial difference in the accessibility profile between *in vitro* CTLs and LCMV-specific *in vivo* CTLs (51). Therefore, despite the similarity in expression pattern of key CTL molecules (52), *in vitro* and *in vivo* CTLs might be generated by differential transcriptional programs, and the contribution of MAZR and Runx3 to these programs might be different between *in vitro* and *in vivo* CTLs. In this regard, while a study from Xue’s group has shown that Runx3-deficient CD8^+^ T cells are more prone to apoptosis upon LCMV infection (17), it remains unclear whether their impaired expansion *in vivo* (**Figures 6A, B**) is due to defects in both proliferation and cell survival as observed *in vitro* (**Figures S2A–E**). CTL subsets are highly heterogeneous and their differentiation depends on the types of pathogens and tumors including differential cytokine milieu (e.g. predominant production of IL-12 and IFN-α, upon Toxoplasma gondii and LCMV infections, respectively) (53–57). One might even speculate that a differential interplay between MAZR and Runx3 is part of the mechanisms underlying the generation of the CTL diversity. Indeed, in “inflammatory” CTLs generated *in vitro* in the presence of IL-12 (58) MAZR and Runx3 regulate the expression of CTL proteins in a different manner (**Figure S7E, F**), compared to CTLs stimulated with anti-CD3/28 “alone” (**Figure 1**). Moreover, it has been postulated that the combinatorial activity of two other transcription factors, T-bet and Blimp1, is important for robust CTL responses against various types of infections and tumor development (59). It might be therefore interesting to test the role of MAZR and Runx3 in CTL induction in other infection models or for cancer immunity.

Finally, our study revealed distinct roles of MAZR and Runx3 for memory T cell subset differentiation. Consistent with a previous study (18), Runx3 is required for the generation of MP subset at the effector phase, and contributes to the establishment of CD8^+^ memory T cells (in particular EM and CM cells). In contrast, MAZR appears to negatively regulate memory T cell differentiation, and its deletion impairs the differentiation of CTLs into TE subset and eventually leads to the enlargement of the EM population. Given that CTLs *in vivo* mainly consist of TE cells, the acquisition of CTL effector function might be tightly linked with naïve-to-TE cell differentiation. Our data suggest that this differentiation process is in part mediated by MAZR. Moreover, since Runx3-deficient CTLs (contain approx. 80% of TE cells) displayed impaired effector function (e.g. impaired Granzyme B expression and reduced proportion of IFNγ^+^TNFα^+^ double producers), this indicates functional alteration of TE cells by loss of Runx3. Therefore, unlike their distinct roles in memory T cells, MAZR and Runx3 might cooperatively promote TE cell differentiation, through regulating different aspects of its differentiation process (i.e. MAZR initiates TE differentiation program, whereas Runx3 mediates their functional maturation). Loss of both MAZR and Runx3 might result in combined defects in the generation of TE cells, leading to further impairment of CTL function *in vivo*. Since our transcriptome analysis identified unique sets of genes regulated by either MAZR or Runx3 (**Figures S3B, C**), this might be linked with their differential regulation of TE cell differentiation (and also their distinct roles for the generation of memory T cells). During the last two decades, sets of transcription factors essential for memory subset differentiation (e.g. TE-versus-MP cell fate decision) have been identified (8–10). Among those, the roles of four transcription factors (T-bet, Blimp1, BCL6, FOXO1) have been recently reassessed with regard to TEM, EM and CM subset differentiation (using the “new” CD127/CD62L-based scheme) (48). This analysis revealed that T-bet and Blimp1 suppress development of EM and CM cells, whereas FOXO1 is required for the generation of both subsets (48). Therefore, these transcription factors exert partially overlapping function of either MAZR or Runx3, and the investigation of their molecular relationship/interaction with MAZR and Runx3 might provide further insight into a transcriptional network underlying memory T cell heterogenicity. In addition, given that EM cells consist of, at least, three transcriptionally distinct subsets (which include recently identified CX3CR1^int^ peripheral memory (PM) cells) (49, 60) and that loss of Runx3 leads to the appearance of atypical KLRG1^–^CD127^–^ or CD62L^+^CD127^–^ memory subset (**Figures 7C–F**), it is of great interest to further characterize the subset composition of the memory T cell pool by loss of MAZR and/or Runx3, utilizing single cell-RNA sequencing combined with high-throughput flow cytometry.

In summary, our study demonstrated that MAZR compensates for loss of Runx3 during CTL differentiation, whereas the two molecules have distinct functions for the generation of memory T cell subset. This highlights a complex interplay between MAZR and Runx3 during CTL/memory T cell differentiation.

## Data Availability Statement

The datasets generated for this study can be found in the Gene Expression Omnibus database, accession number: GSE129772.

## Ethics Statement

The animal study was reviewed and approved by Federal Ministry for Education, Science and Research, Vienna, Austria (GZ:BMWFW-66.009/0011-WF/V/3b/2017, BMWFW-66.009/0343-V/3b/2019).

## Author Contributions

AG, RR, CT and DH performed experiments and analyzed the data. CV, KK and AB helped with infection experiments and measured viral titers. TF, AL and LE performed bioinformatic analysis. TP performed next generation sequencing under the supervision of CB. RB provided mice. WE designed the research and wrote the manuscript. SS designed the research, performed experiments, analyzed the data and wrote the manuscript. All authors contributed to the article and approved the submitted version.

## Funding

The work in the group of SS was supported by Austrian Science Fund (FWF) projects: P23669 and P27747. AG was in part supported by The L’ORÉAL Austria Fellowship Programm organized by L’ORÉAL Austria, the Austrian UNESCO Commission, the Austrian Academy of Sciences and the Federal Ministry of Education, Science and Research. The work in the group of WE was supported by Austrian Science Fund (FWF) projects: P29790 and by the FWF and Medical University of Vienna doctoral programs (DK W1212) “Inflammation and Immunity” and (DOC 32) “TissueHome”. The work in the laboratory of AB was supported by Austrian Science Fund (FWF) projects: P23991, P25360 and P30047, and by an ERC Starting Grant (European Union’s Horizon 2020 research and innovation program, grant agreement numbers 677006). KK was supported by the DOC Fellowship Programme of the Austrian Academy of Sciences. The work in the laboratory of LE was supported by the European Union’s Horizon 2020 research and innovation programme ENLIGHT-TEN under the Marie Sklodowska-Curie grant agreement No.: 675395. LE reports grants from the European Research Council ERC (677943), European Union’s Horizon 2020 research and innovation programme (675395), Academy of Finland (296801, 304995, 310561 and 313343), Juvenile Diabetes Research Foundation JDRF (2-2013-32), Tekes – the Finnish Funding Agency for Innovation (1877/31/2016), Sigrid Juselius Foundation, Turku Graduate School (UTUGS), University of Turku, Åbo Akademi University, Biocenter Finland and ELIXIR Finland node. RB was supported by the Intramural Research Program of the National Cancer Institute, Center for Cancer Research, National Institutes of Health.

## Conflict of Interest

The authors declare that the research was conducted in the absence of any commercial or financial relationships that could be construed as a potential conflict of interest.
