# Supplementary material for: Complex Interplay Between MAZR and Runx3 Regulates the Generation of Cytotoxic T Lymphocyte and Memory T Cells
Source: Front Immunol. 2021 Mar 17;12:535039. doi: 10.3389/fimmu.2021.535039 (PMC8010151; doi:10.3389/fimmu.2021.535039)
Supplement: Supplementary file 1 [file DataSheet_1.pdf]

frontiers

Supplementary Material

Alexandra Franziska Gülich[1†], Ramona Rica[1†], Caroline Tizian[1,6], Csilla Viczenczova[2], Kseniya Khamina[2,7], Thomas Faux[3], Daniela Hainberger[1], Thomas Penz[2], Remy Bosselut[5], Christoph Bock[2,4], Asta Laiho[3], Laura L. Elo[3], Andreas Bergthaler[2], Wilfried Ellmeier[1], Shinya Sakaguchi[1*]

[1]Division of Immunobiology, Institute of Immunology, Center for Pathophysiology, Infectiology and Immunology, Medical University of Vienna, 1090 Vienna, Austria

[2]CeMM Research Center for Molecular Medicine of the Austrian Academy of Sciences, 1090 Vienna, Austria

[3]Turku Bioscience Centre, University of Turku and Åbo Akademi University, 20520 Turku, Finland

[4]Institute of Artificial Intelligence and Decision Support, Center for Medical Statistics, Informatics, and Intelligent Systems, Medical University of Vienna, Vienna, Austria

[5]Laboratory of Immune Cell Biology, Center for Cancer Research, National Cancer Institute, National Institutes of Health, Bethesda, MD 20892, USA

[6]Present address: Laboratory of Innate Immunity, Department of Microbiology, Infectious Diseases and Immunology, Charité-Universitätsmedizin Berlin, Campus Benjamin Franklin, Hindenburgdamm 30, 12203 Berlin, Germany

[7]Present address: TAmiRNA GmbH, 1190 Vienna, Austria

†These authors have contributed equally to this work and share first authorship

***Correspondence:**

Dr. Shinya Sakaguchi

shinya.sakaguchi@meduniwien.ac.at

Supplementary Figures

Figure S1

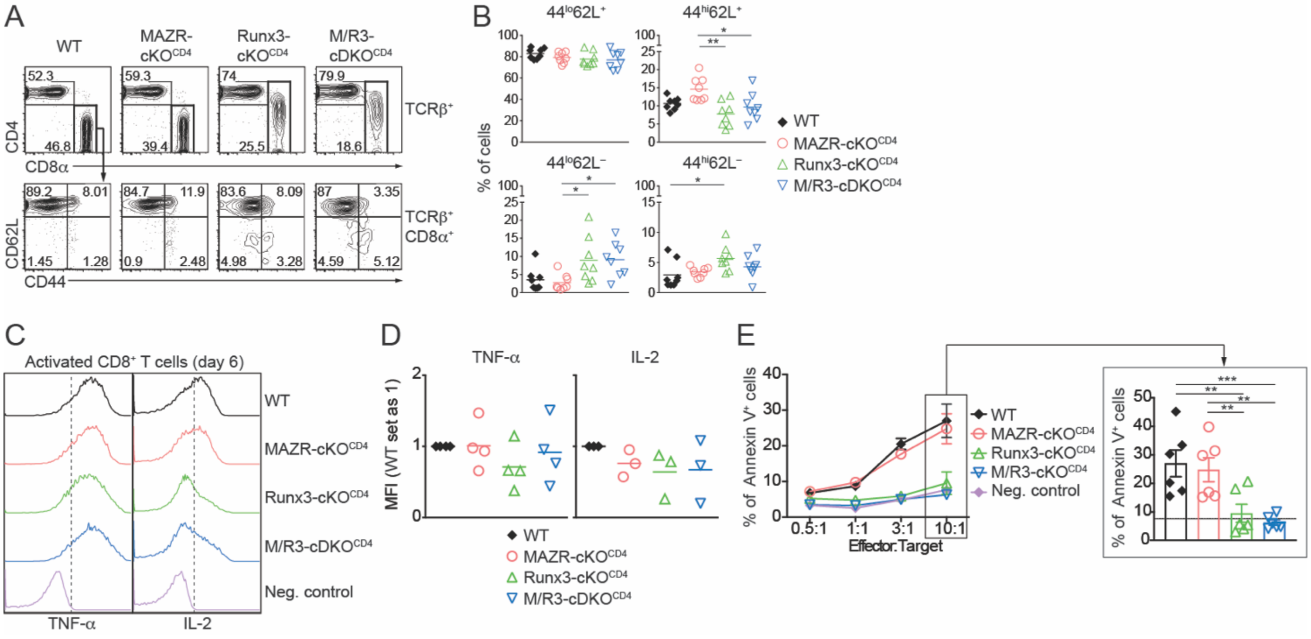

Figure S1. The deletion of MAZR has no impact on cytokine production and cytotoxic activity of CTLs. **(A)** Flow cytometric analysis showing CD4 and CD8α expression in TCRβ$^+$ splenocytes (upper panel) as well as CD62L and CD44 expression (lower panel) in TCRβ$^+$CD8α$^+$ splenocytes of the mutant mice. Numbers indicates percentages in the respective regions. **(B)** Diagrams showing the percentages of CD44^{lo}CD62L$^+$ (upper left), CD44^{hi}CD62L$^+$ (upper right), CD44^{lo}CD62L$^-$ (lower left) and CD44^{hi}CD62L$^+$ (lower right) subsets in TCRβ$^+$CD8α$^+$ splenocytes of the mutant mice. **(C)** Histograms showing TNF-α and IL-2 expression in the mutant CTLs (6 days after *in vitro* anti-CD3/28 stimulation). WT CTLs without restimulation were included as a negative control. Vertical dotted lines indicate populations expressing the respective proteins. **(D)** Diagrams showing the relative mean fluorescence intensity (MFI) of indicated cytokines in the mutant CTLs (the values of WT cells set as 1). **(E)** Redirected *in vitro* cytotoxicity assay of MAZR and/or Runx3-deficient CTLs (see Methods section for the detailed experimental procedure). The left diagram shows the cytolytic activity of *in vitro* differentiated CTLs with the indicated genotypes (i.e. the proportion of apoptotic P815-GFP$^+$ target cells treated with anti-CD3 antibody). WT cells co-cultured with antibody-untreated target cells were provided as a negative control. Values are represented as ± standard error. The right diagram shows the cytotoxic activity of the mutant CTLs at the effector-to-target ratio of 10:1 (outlined at left). **(B,D,E)** Each dot represents one mouse. The horizontal bars indicate the mean. For the comparison of values between four groups **(B,E)** and three mutant groups **(D)**, a one-way ANOVA analysis followed by Tukey's multiple-comparison test was performed. The *p*-values were defined as following: *, $p < 0.05$; **, $p < 0.01$; ***, $p < 0.001$. For the comparison of the relative MFI values between WT (set as 1) and each mutant group **(D)**, a one-sample t-test was performed. No comparison between two groups reached a statistically significant level (i.e. $p < 0.05$). Data are representative **(A,C)** or show the summary **(B,D,E)** of 4-8 mice analyzed in 4 independent experiments.

Figure S2

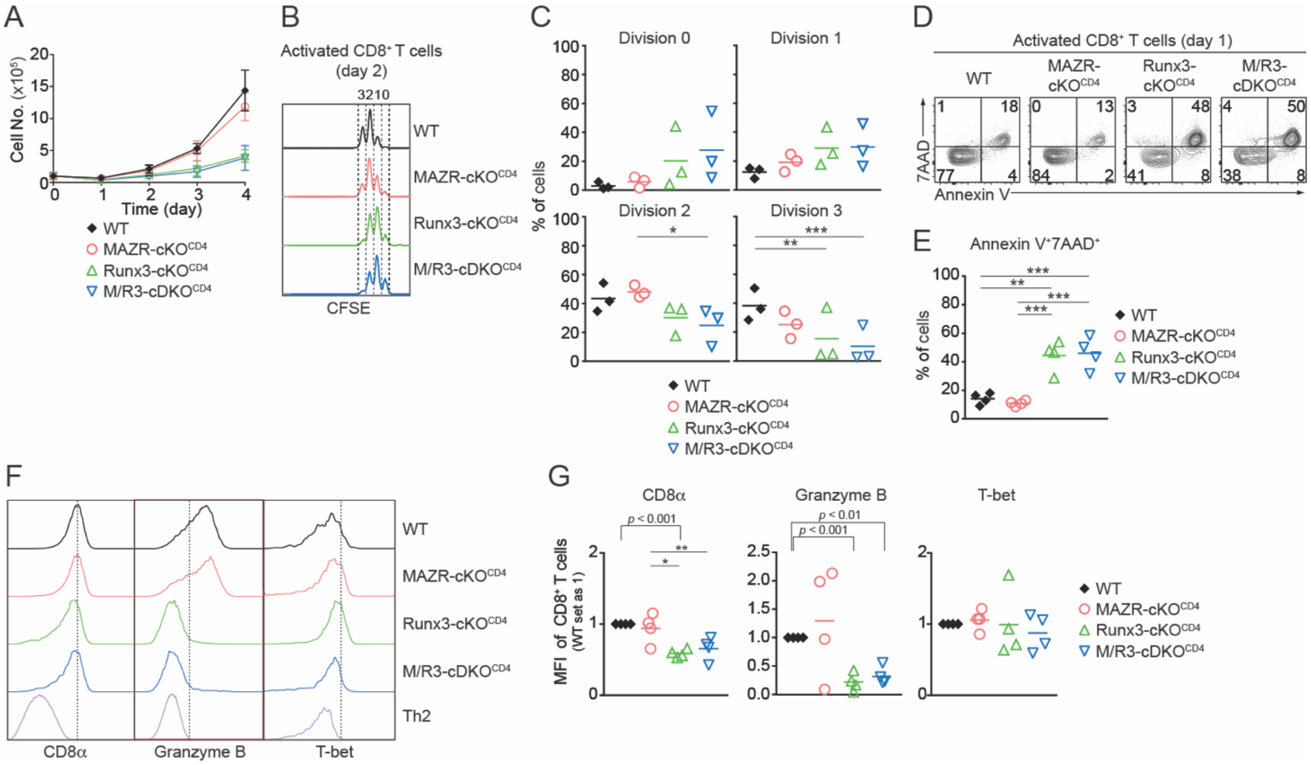

Figure S2. Phenotypic analysis of MAZR- and/or Runx3-deficient CD8$^+$ T cells during during the early phase of CTL differentiation. **(A)** Diagram showing the growth curves of mutant CD8$^+$ T cells upon *in vitro* anti-CD3/CD28 stimulation. Error bars indicate SD. **(B)** Histograms showing the cell divisions of CFSE-labeled mutant CD8$^+$ T cells (2 days after anti-CD3/CD28 stimulation). The numbers of divisions are indicated above histograms. **(C)** Diagrams showing the percentages of CD8$^+$ T cells in each division upon anti-CD3/CD28 stimulation (day 2). **(D)** Flow cytometric analysis showing Annexin V and 7AAD expression in anti-CD3/CD28-stimulated mutant CD8$^+$ T cells (day 1). Numbers indicate the percentage of cells in the respective regions. **(E)** Diagrams showing the percentage of Annexin V$^+$7AAD$^+$ population within anti-CD3/CD28-stimulated mutant CD8$^+$ T cells (day 1). **(F)** Histograms showing the expression patterns of the CD8α, Granzyme B and T-bet in CD8$^+$ T cells of the indicated genotypes at day 3 of *in vitro* activation. As negative controls for stainings, activated Th2 cells from WT mice were included. Vertical dotted lines indicate the peak of CD8α expression in WT CTLs as well as populations expressing Granzyme B and T-bet based on the negative controls. **(G)** Diagrams displaying the relative mean fluorescence intensity (MFI) of indicated proteins in the mutant CTLs (the values of WT cells set as 1). **(C,E,G)** Each dot represents one mouse. The horizontal bars indicate the mean. For the comparison of values between four groups **(C,E)** and three groups of the mutant mice **(G)**, a one-way ANOVA analysis followed by Tukey's multiple-comparison test was performed. The *p*-values were defined as following: *, $p < 0.05$; **, $p < 0.01$; ***, $p < 0.001$. For the comparison of the relative MFI values between WT (set as 1) and each mutant group **(G)**, a one-sample t-test was performed. The *p*-values are indicated above the diagrams. Data are representative **(B,D,F)** or show the summary **(A,C,E,G)** of 3-4 mice analyzed in 3-4 independent experiments.

Figure S3

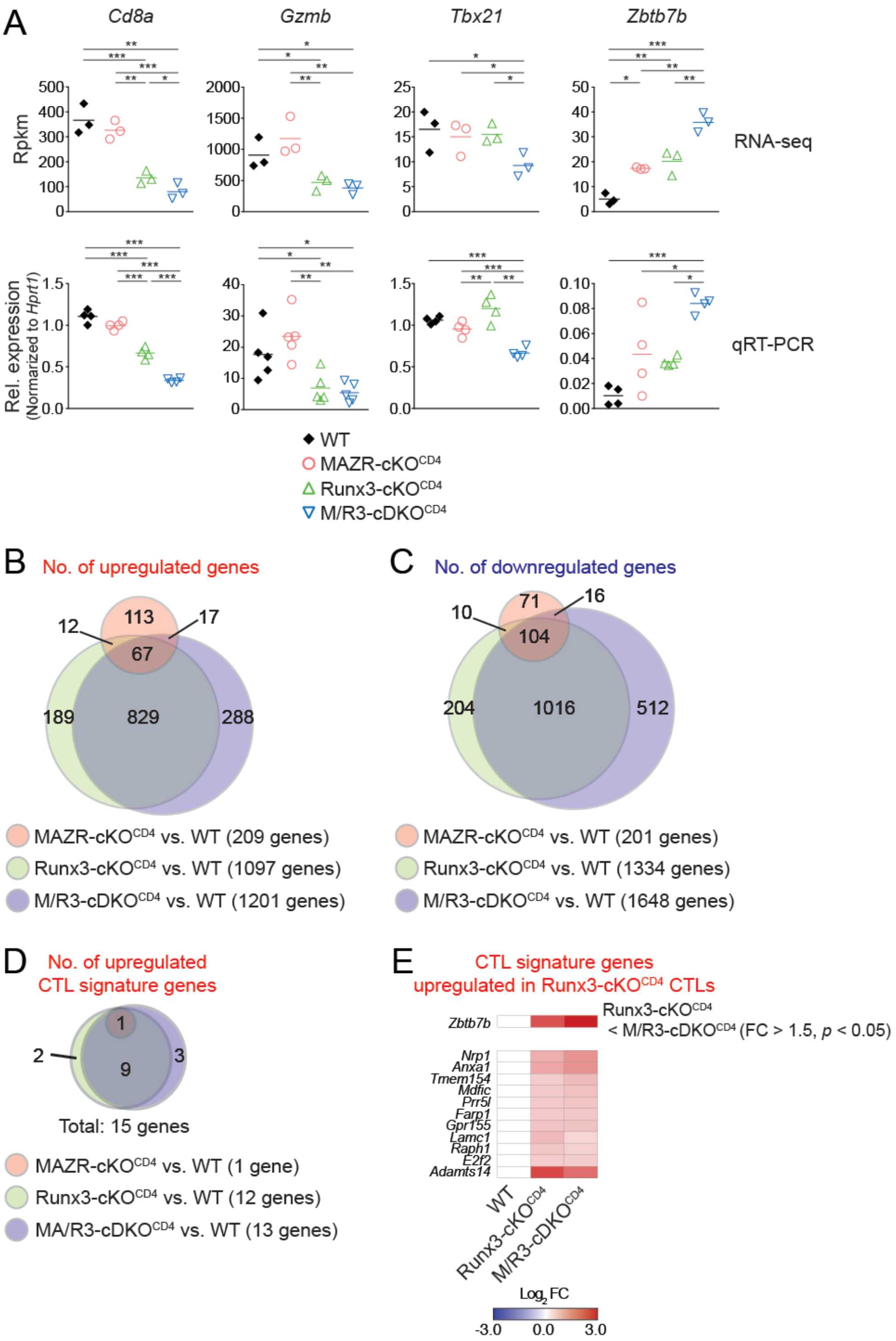

Figure S3. Transcriptome analysis of MAZR- and/or Runx3-deficient CTLs. **(A)** Validation of the results obtained from transcriptome analysis by qRT-PCR. Diagrams displaying the expression of the indicated genes in the mutant CTLs, as determined by transcriptome analysis (upper panel, values shown as reads per kilobase of transcript per million mapped reads; Rpkm) as well as by qRT-PCR (lower panel, values shown as relative expression levels normalized to the one of the *Hprt1* gene). Each symbol indicates 1 biological sample. *, $p < 0.05$; **, $p < 0.01$; ***, $p < 0.001$ (one-way ANOVA analysis followed by Tukey's multiple-comparison). **(B)** Venn diagrams displaying the overlaps of upregulated genes in the individual mutant CTLs, compared to WT cells. **(C)** Venn diagrams displaying the overlaps of downregulated genes in the individual mutant CTLs, compared to WT cells. **(D)** Venn diagram displaying the overlaps of upregulated "CTL signature" genes in the individual mutant CTLs compared to WT cells. **(E)** Heatmap showing fold change (FC; log2 transformed) differences of 12 CTL signature genes upregulated in R3-cDKO^{CD4} CTLs compared to WT cells. The expression patterns of the genes in R3-cDKO^{CD4} and M/R3-cDKO^{CD4} CTLs are shown (mean expression levels in WT CTLs were set as 1). The *Zbtb7b* gene (encoding ThPOK) displays a further upregulation in M/R3-cDKO^{CD4} CTLs (upper panel; based on a threshold of FC > 1.5 and *p*-value < 0.05), and the rest 11 genes are presented in the lower panel. Gene names are indicated at the left. **(B,C,D)** The numbers of genes in the respective regions are indicated in the diagrams.

Figure S4

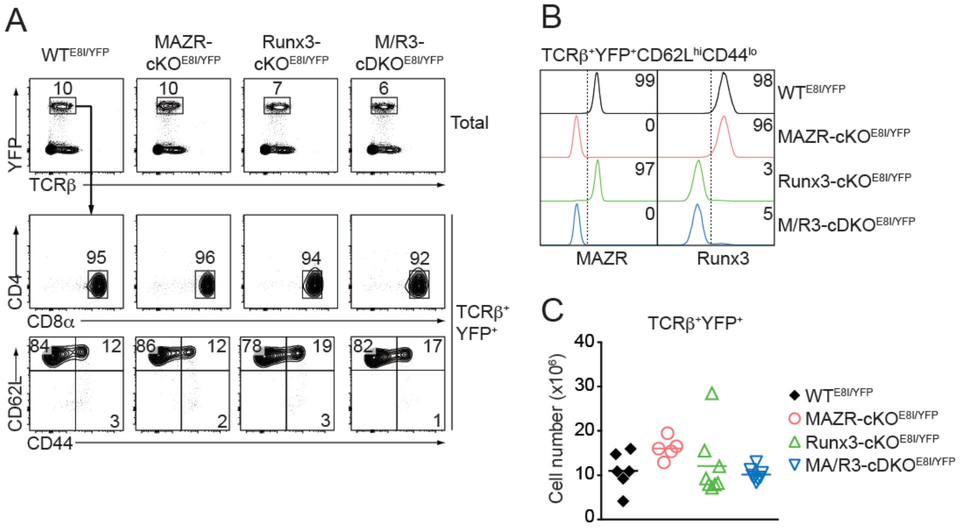

Figure S4. Phenotypic analysis of peripheral CD8$^+$ T cells in MAZR-cKO$^{E8I/YFP}$, Runx3-cKO$^{E8I/YFP}$ and M/R3-cDKO$^{E8I/YFP}$ mice. **(A)** Flow cytometric analysis showing YFP and TCRβ expression in total splenocytes (upper panel), CD4 and CD8α (middle panel) as well as CD62L and CD44 expression (lower panel) in TCRβ$^+$YFP$^+$ splenocytes of the mutant mice. Numbers indicate percentages in the respective regions. **(B)** Histograms showing MAZR (left panel) and Runx3 (right panel) expression in TCRβ$^+$YFP$^+$CD62L^{hi}CD44^{lo} splenocytes of the mutant mice. Numbers indicate the percentages of MAZR$^+$ (left panel) and Runx3$^+$ (right panel) cells. **(C)** Diagram showing the numbers of TCRβ$^+$YFP$^+$ splenocytes of the mutant mice. A one-way ANOVA analysis was performed. No comparison between two groups reached a statistically significant level (i.e. $p < 0.05$). **(A,C)** Data are representative **(A)** or show the summary **(C)** of 5-8 mice analyzed in 3 independent experiments. **(B)** Data are representative of 3 mice analyzed in 2 independent experiments.

Figure S5

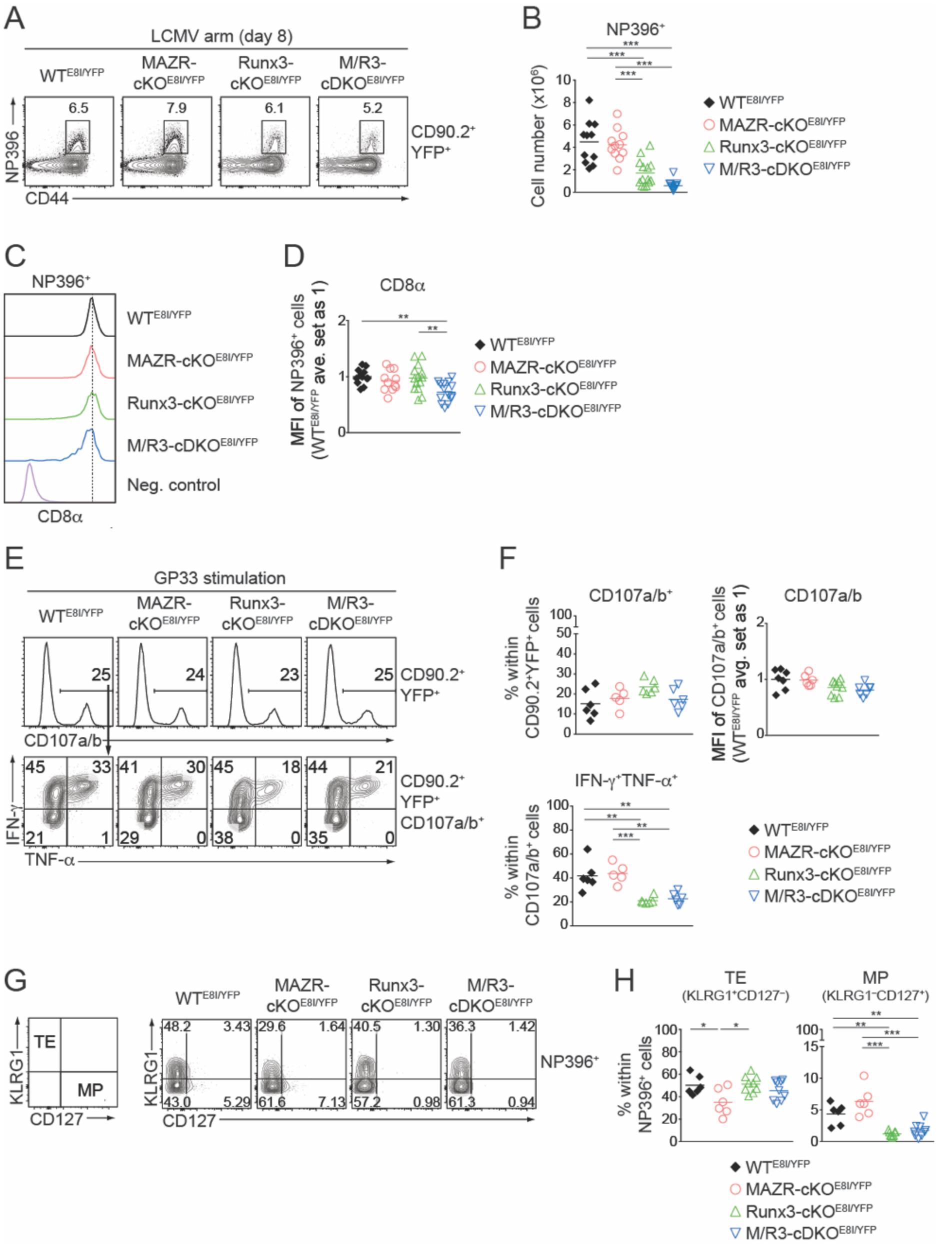

Figure S5. Phenotypic analysis of NP396 peptide-specific mutant CTLs as well as CD107 surface externalization and cytokine production of the mutant CTLs upon GP33 peptide restimulation. **(A)** Flow cytometric analysis showing CD44 expression and H2-D^{b}/NP396-404 dextramer (NP396) staining of CD90.2^{+}YFP^{+} splenocytes isolated from mice of the indicated genotypes (eight days after LCMV Armstrong infection). **(B)** Diagram showing the cell numbers of CD90.2^{+}YFP^{+}NP396^{+} splenocytes of the indicated genotypes. **(C)** Histograms showing CD8α expression on NP396^{+} splenocytes of the mutant mice. Naïve WT$^{E8I/YFP}$ CD4^{+} T cells were included as a negative control for staining. **(D)** Diagram showing the mean fluorescence intensity (MFI) of CD8α expression on NP396^{+} splenocytes of the mutant mice. The average value of WT$^{E8I/YFP}$ cells was set as 1. **(E)** Flow cytometric analysis showing CD107a/b surface externalization in CD90.2^{+}YFP^{+} splenocytes (upper panel) and IFN-γ and TNF-α expression in CD107a/b^{+}CD90.2^{+}YFP^{+} splenocytes (lower panel) of the mutant mice. **(F)** Diagrams showing the percentage of CD107a/b^{+} population (upper left panel) within CD90.2^{+}YFP^{+} splenocytes, the mean fluorescence intensity (MFI) of CD107a/b expression on CD107a/b^{+} population (upper right panel) and the percentage of IFN-γ^{+}TNF-α^{+} population within CD90.2^{+}YFP^{+}CD107a/b^{+} splenocytes (lower panel) of the mutant mice. For MFI the average value of WT$^{E8I/YFP}$ cells was set as 1. **(G)** Flow cytometric analysis showing KLRG1 and CD127 expression in CD90.2^{+}YFP^{+}NP396^{+} (NP396^{+}) splenocytes of the indicated genotypes (eight days after LCMV Armstrong infection). The panel on the left indicates the definition of individual subsets: KLRG1^{+}CD127^{-} terminal effector (TE) and KLRG1^{-}CD127^{+} memory precursor (MP) cells. (**H**) Diagrams showing the percentage of the indicated subsets (identified as shown in (**G**)) within NP396^{+} splenocytes of the indicated genotypes. **(A,E,G)** Numbers indicate percentages of the respective regions. **(B,D,F,H)** Each dot represents one mouse. The horizontal bars indicate the mean. *, $p < 0.05$; **, $p < 0.01$; ***, $p < 0.001$ (one-way ANOVA analysis followed by Tukey's multiple-comparison). Data are representative (**A,E,G**) or show the summary (**B,D,F,H**) of 6-14 mice analyzed in 6-8 independent experiments.

Figure S6

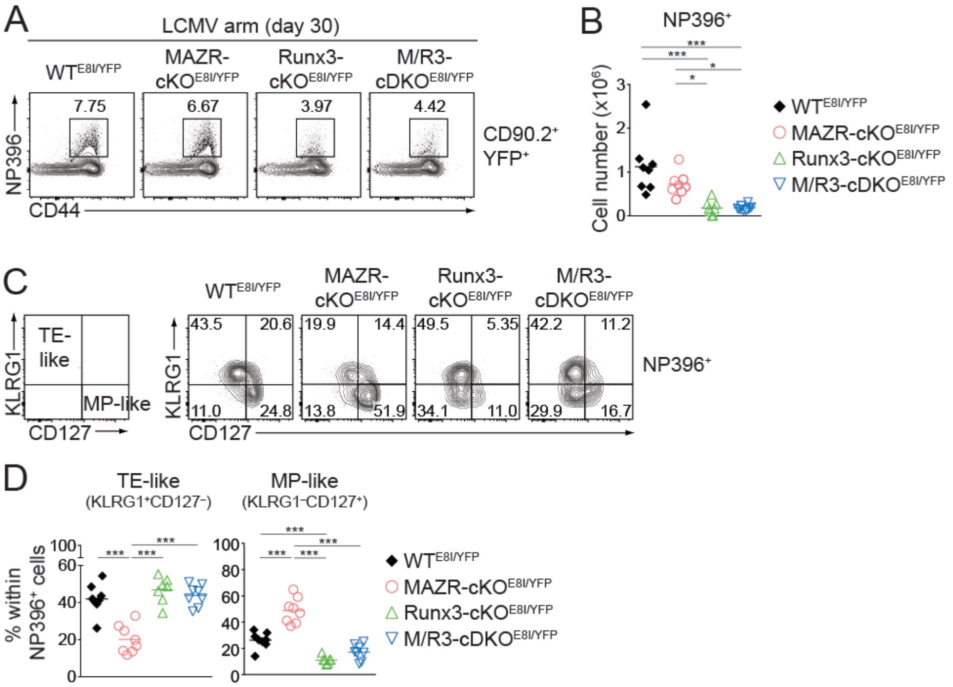

Figure S6. Altered memory T cell subset differentiation in the absence of MAZR and/or Runx3 **(A)** Flow cytometric analysis showing CD44 expression and H2-D^{b}/NP396-404 dextramer (NP396) staining of CD90.2^{+}YFP^{+} splenocytes isolated from mice of the indicated genotypes (thirty days after LCMV Armstrong infection). **(B)** Diagrams showing the cell numbers of CD90.2^{+}YFP^{+}NP396^{+} splenocytes of the indicated genotypes. **(C)** Flow cytometric analysis showing KLRG1 and CD127 expression in NP396^{+} splenocytes of the indicated genotypes. The panel on the left indicates the definition of individual subsets: KLRG1^{+}CD127^{-} terminal effector-like (TE-like) and KLRG1^{-}CD127^{+} memory precursor-like (MP-like) cells. **(D)** Diagrams showing the percentage of the indicated subsets (identified as shown in **(C)**) within NP396^{+} splenocytes of the indicated genotypes. **(A,C)** Numbers indicate percentages of the respective regions. **(B,D)** Each dot represents one mouse. The horizontal bars indicate the mean. *, $p < 0.05$; **, $p < 0.01$; ***, $p < 0.001$ (one-way ANOVA analysis followed by Tukey's multiple-comparison). Data are representative (**A,C**) or show the summary (**B,D**) of 7-8 mice analyzed in 2 independent experiments.

Figure S7

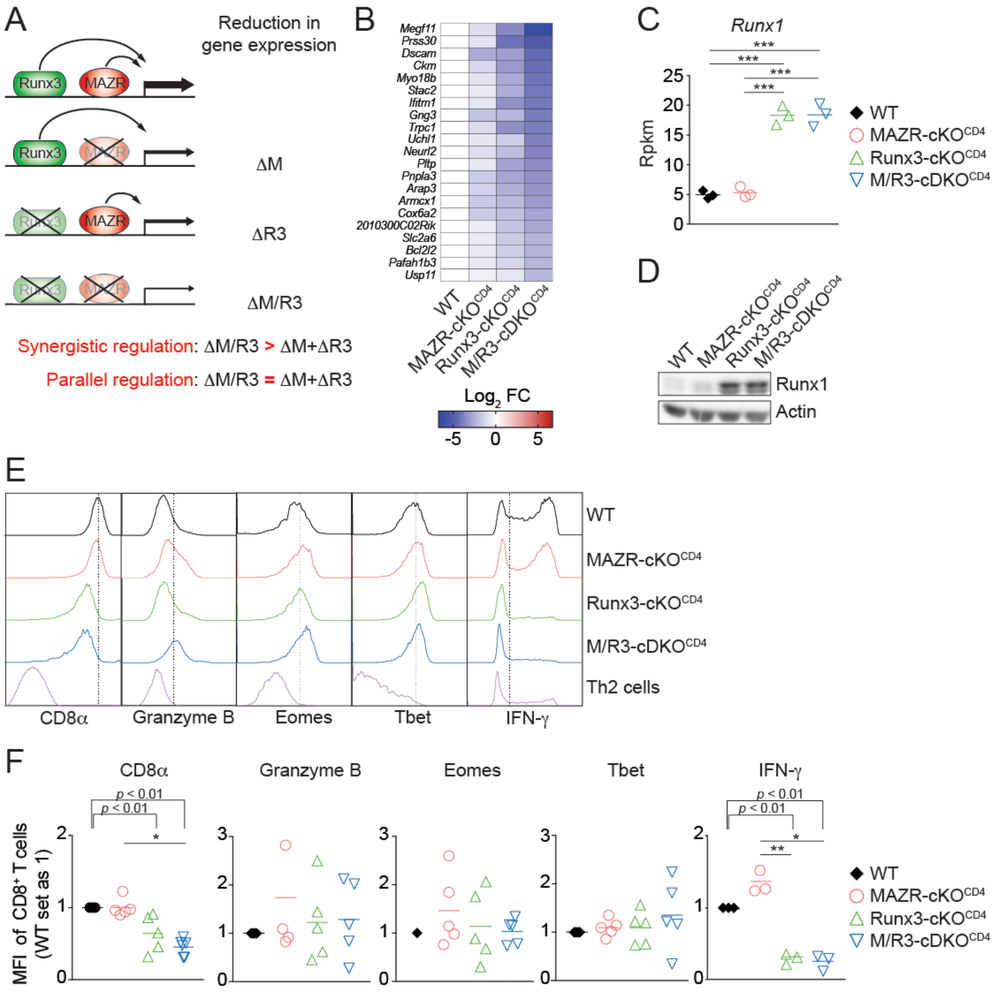

Figure S7. Model of synergistic and parallel gene regulation mediated by MAZR and Runx3, Runx1 expression in MAZR- and/or Runx3-deficient CTLs and phenotypic analysis of the mutant CTLs differentiated *in vitro* in the presence of IL-12. **(A)** Simplified scheme showing synergistic and parallel regulation of gene expression by MAZR and Runx3. Synergistic regulation: the fold-change difference of a target gene in the absence of MAZR/Runx3 (ΔM/R3) is higher than the sum of individual differences (ΔM+ΔR3). Parallel regulation: the fold-change difference in the absence of MAZR/Runx3 (ΔM/R3) is similar to the sum of individual differences (ΔM +ΔR3). Compensation (not shown): the effect of MAZR deletion is only seen in the absence of Runx3. **(B)** Heatmap showing fold change (FC; log2 transformed) differences of 21 genes synergistically repressed by MAZR and Runx3. Among 104 genes downregulated in all the three mutant CTLs (Figure S3C)), genes that display greater FC in M/R3-cDKO^{CD4} CTLs than additive FC of the single deletions were shown. Gene names are indicated at the left. **(C)** Diagram showing expression (reads per kilobase of transcript per million mapped reads; Rpkm) of *Runx1* in CTLs of the indicated genotypes as determined by RNA-Seq. Each symbol indicates 1 biological sample. The horizontal bars indicate the mean. **(D)** Immunoblot showing Runx1 expression in CTLs of the indicated genotypes. Actin was used as loading control. **(E)** Histograms show expression of the indicated proteins in the mutant CTLs five days after *in vitro* activation in the presence of IL-12 (2 ng/ml). As negative staining controls WT Th2 cells were included. Vertical dotted lines indicate the peak of CD8 expression in WT CTLs as well as populations expressing respective proteins based on the negative controls. **(F)** Diagrams showing the relative mean fluorescence intensity (MFI) of indicated proteins in the mutant CTLs treated with IL-12 (the values of WT cells set as 1). Each dot represents one mouse. The horizontal bars indicate the mean. **(C,F)** For the comparison of values between four groups **(C)** and three groups of the mutant mice **(F)**, a one-way ANOVA analysis followed by Tukey's multiple-comparison test was performed. The *p*-values were defined as following: *, $p < 0.05$; **, $p < 0.01$; ***, $p < 0.001$. For the comparison of the relative MFI values between WT (set as 1) and each mutant group **(F)**, a one-sample t-test was performed. The *p*-values are indicated above the diagrams. Data are representative **(D,E)** or show the summary **(F)** of 2 **(D)** and 5 **(E,F)** mice analyzed in 2 **(D)** and 5 **(E,F)** independent experiments.

Supplementary Tables

Table S1. Antibodies used in this study

Antigen	Clone or Catalog number (#)	Company
CD107a	1D4B	Biolegend
CD107b	M3/84	Biolegend
CD127	A7R34	Biolegend
CD4	RM4-5	Thermo Fisher Scientific
CD44	IM7	Biolegend
CD62L	MEL-14	Biolegend
CD8α	53-6.7	Thermo Fisher Scientific
CD90.2	53-2.1	Biolegend
CXCR5	L138D7	Biolegend
Eomes	Dan11mag	Thermo Fisher Scientific
IFN-γ	XMG1.2	BD Biosciences
IL-2	JES6-5H4	BD Biosciences
Granzyme B	GB11	BD Biosciences
MAZR/PATZ1	D-5	Santa Cruz Biotechnology
Mouse IgG_1	RMG1-1	Biolegend
Perforin	S16009A	Biolegend
Runx3	R3-5G4	BD Biosciences
T-bet	4B10	Biolegend
TCRβ	H57-597	Thermo Fisher Scientific
TNF-α	MP6-XT22	Thermo Fisher Scientific
